# A synthetic porphyrin as an effective dual antidote against carbon monoxide and cyanide poisoning

**DOI:** 10.1073/pnas.2209924120

**Published:** 2023-02-21

**Authors:** Qiyue Mao, Xuansu Zhao, Akiko Kiriyama, Shigeru Negi, Yasutaka Fukuda, Hideki Yoshioka, Akira T. Kawaguchi, Roberto Motterlini, Roberta Foresti, Hiroaki Kitagishi

**Affiliations:** ^a^Department of Molecular Chemistry and Biochemistry, Faculty of Science and Engineering, Doshisha University, Kyotanabe, Kyoto 610-0321, Japan; ^b^Department of Fire Engineering, Building Research Institute, Tsukuba, Ibaraki 305-0802, Japan; ^c^Faculty of Pharmaceutical Science, Doshisha Women’s College of Liberal Arts, Kyotanabe, Kyoto 610-0395, Japan; ^d^Tsukuba Building Research and Testing Laboratory, Center for Better Living, Tsukuba, Ibaraki 305-0802, Japan; ^e^Department of Architecture, Graduate School of Engineering, The University of Tokyo, Hongo, Bunkyo-ku, Tokyo 113-8656, Japan; ^f^Department of Innovative Medical Science Tokai University, Isehara, Kanagawa 259-1193, Japan; ^g^University Paris Est Creteil, INSERM, Institut Mondor de Recherche Biomédicale (IMRB), Creteil F-94010, France

**Keywords:** carbon monoxide, cyanide, fire gas, porphyrin, supramolecular chemistry

## Abstract

Simultaneous poisoning by carbon monoxide (CO) and hydrogen cyanide is the major cause of mortality in fire gas accidents. Here, we report on the invention of an injectable antidote against CO and cyanide (CN^–^) mixed poisoning. The solution contains four compounds: iron(III)porphyrin (Fe^III^TPPS, F), two methyl-β-cyclodextrin (CD) dimers linked by pyridine (Py3CD, P) and imidazole (Im3CD, I), and a reducing agent (Na_2_S_2_O_4_, S). When these compounds are dissolved in saline, the solution contains two synthetic heme models including a complex of F with P (hemoCD-P) and another one of F with I (hemoCD-I), both in their iron(II) state. hemoCD-P is stable in its iron(II) state and captures CO more strongly than native hemoproteins, while hemoCD-I is readily autoxidized to its iron(III) state to scavenge CN^–^ once injected into blood circulation. The mixed solution (hemoCD-Twins) exhibited remarkable protective effects against acute CO and CN^–^ mixed poisoning in mice (~85% survival vs. 0% controls). In a model using rats, exposure to CO and CN^–^ resulted in a significant decrease in heart rate and blood pressure, which were restored by hemoCD-Twins in association with decreased CO and CN^–^ levels in blood. Pharmacokinetic data revealed a fast urinary excretion of hemoCD-Twins with an elimination half-life of 47 min. Finally, to simulate a fire accident and translate our findings to a real-life scenario, we confirmed that combustion gas from acrylic cloth caused severe toxicity to mice and that injection of hemoCD-Twins significantly improved the survival rate, leading to a rapid recovery from the physical incapacitation.

## Significance

In fire accidents, highly toxic gases such as carbon monoxide (CO) and hydrogen cyanide (HCN) are simultaneously generated during the combustion of building materials. When inhaled, these two gases strongly bind to hemoglobin, cytochromes, and other hemes in the living organisms, thus inhibiting aerobic respiration. To date, there is no therapeutic approach to overcome simultaneous poisoning with CO and HCN. Here, we invented a synthetic heme model compound (hemoCD-Twins) to provide emergency life-saving treatment. This compound captures CO and cyanide inhaled into animals with a single injection and is rapidly eliminated via urinary excretion. With an immediate antidotal effect, high degree of safety, and storage stability, hemoCD-Twins has great potential to be a ready-to-use antidote against fire gas poisoning.

Fire accidents frequently occur around the world and the consequent inhalation of combustion gases represents the leading cause of death under these circumstances (1–10). Typically, combustion gases consist, among others, of carbon dioxide, water (H_2_O), carbon monoxide (CO), hydrogen cyanide (HCN), hydrochloric acid, nitrogen oxide, and sulfur oxide. Their amounts depend on the materials of combustion (3–10) and, among them, CO and HCN are the most dangerous due to their severe toxic effects in humans (3–4, 5–15). CO is generated during incomplete combustion of carbon materials while HCN derives from carbon- and nitrogen-containing materials. Thus, CO and HCN gases are simultaneously released when synthetic materials such as plastics, acrylic cloths, and urethanes are burned in buildings. Once inhaled, CO strongly binds to ferrous iron(II) heme proteins such as hemoglobin (Hb) in erythrocytes and myoglobin in muscles (16–19), thus compromising oxygen (O_2_) transport/storage in the blood circulation and tissues. On the contrary, HCN binds as cyanide ion (CN^–^) to ferric iron(III) heme, preferentially targeting cytochrome *c* oxidase (C*c*O) in mitochondria and interrupting O_2_-dependent energy production (11, 20–22). Likewise, the toxic effects of CO on mitochondrial respiration are ascribed to its ability to bind to cytochrome *c* oxidase (23–25). Therefore, both CO and HCN can inhibit aerobic respiration mediated by several heme proteins leading to anoxia-induced lethal toxicity. As the molecular mechanisms of CO and HCN toxicity are closely related, additional or synergistic deleterious effects can be expected when these two gases are produced and inhaled at the same time (26–31). Therapeutic strategies to treat either CO or HCN poisoning have been developed independently (11, 13, 22, 32–37). However, to our knowledge, there is no medical intervention at present to neutralize simultaneously CO and HCN with an effective treatment in vivo.

**Fig. 1.**
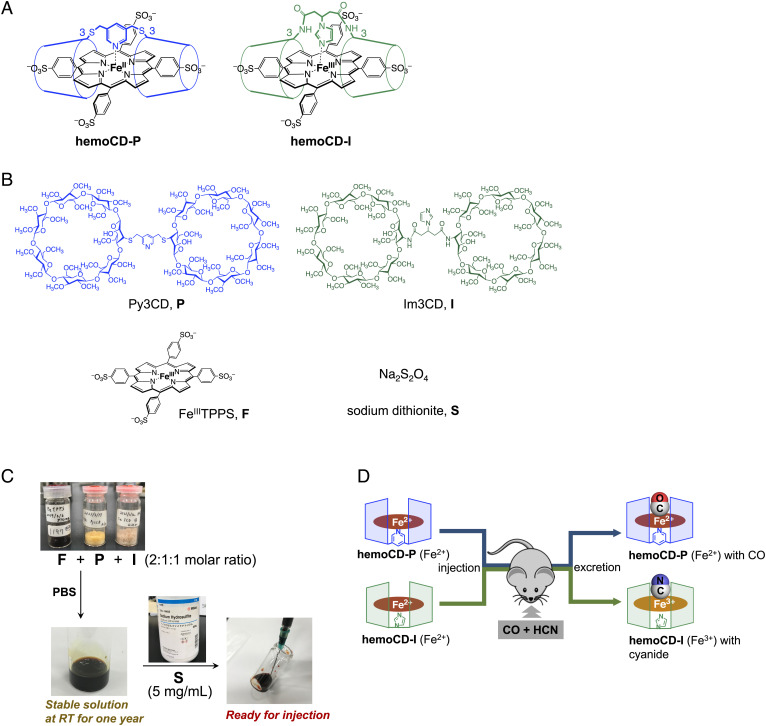
hemoCD-Twins as an antidote system against CO and CN^–^ mixed poisoning. (*A*) Biomimetic heme protein model compounds hemoCD-P and hemoCD-I that are used as CO and CN^–^ scavengers in vivo. (*B*) Chemical structures of the four components P, I, F, and S contained in hemoCD-Twins. (*C*) Preparation of hemoCD-Twins. Three powder compounds, F, P, and I, were dissolved in PBS in a 2:1:1 molar ratio, where the solution contains hemoCD-P and hemoCD-I in ferric iron(III) states. The solution is stable and storable at room temperature. The reducing agent S is added just before use. (*D*) Schematic illustration for the simultaneous removal of CO and CN^–^ by a single injection of hemoCD-Twins in vivo. CO and CN^–^ are removed from living organisms and excreted in the urine with hemoCD-P and hemoCD-I in ferrous iron(II) and ferric iron(III) complexes, respectively.

In the present study, we have developed an injectable antidote for CO and HCN mixed poisoning. This agent contains supramolecular compounds, termed **hemoCD**s, composed of a water-soluble iron(II/III)porphyrin and two cyclodextrin (CD) dimers. Based on our previous research on the synthesis and characterization of heme protein model structures (38–40), the gas-binding ability and the redox status of the iron center of the porphyrin can be regulated by the linker structure of the CD dimers englobing the porphyrin. The porphyrin complexed with a CD dimer having a pyridine linker (hemoCD-P, Fig. 1 *A*, *Left*) is stable in the iron(II) state in vivo and shows much higher CO-binding affinity compared to the affinities reported for native heme proteins (41–45). On the contrary, the porphyrin complexed with a CD dimer having an imidazole linker (hemoCD-I, Fig. 1 *A*, *Right*) is stable in the iron(III) state and shows a higher binding affinity to CN^–^ than native ferric met-hemoglobin (met-Hb) (46, 47). These two complexes do not bind to plasma proteins in the circulation when injected separately and are quickly excreted in urines without any chemical decomposition. Therefore, these compounds have the ability to capture either CO or CN^–^ to their iron(II/III) centers in vivo and expel these toxic ligands from the organism (41–47). Here, we have developed hemoCD-Twins that contains both hemoCD-P and hemoCD-I in saline for simultaneous removal of CO and HCN in vivo. This paper describes the primary pharmacological properties of hemoCD-Twins and its detoxification effects in animals intoxicated with CO and CN^–^. To simulate a real-life fire accident, the burning of acrylic cloth with consequent release of combustion gases was also used as an experimental approach for antidotal tests. We found that hemoCD-Twins exhibits the following features: 1) The synthetic compounds are storable at room temperature over a year thanks to their chemical stability; 2) the solution can be quickly prepared without the need of special handlings; 3) a single administration of hemoCD-Twins shows immediate dual antidotal effect against CO and CN^–^; and 4) the injected solution is quickly eliminated from the body without significant side effects. We are envisioning a scenario whereby a person who is accidentally exposed to fire gases containing CO and HCN can be promptly treated at the site by infusion with hemoCD-Twins.

## Results

### Preparation and Chemical Characterization of hemoCD-Twins.

The solution of hemoCD-Twins as an injectable antidote was prepared by dissolving four compounds: 5,10,15,20-tetrakis(4-sulfonatophenyl)porphinatoiron(II/III) (FeTPPS or F), per-*O*-methylated β-CD dimers having pyridine (Py3CD or P) and imidazole linkers (Im3CD or I), and sodium dithionite (S) in phosphate-buffered saline (PBS) (Fig. 1*B*) (abbreviation list is shown in *SI Appendix*). These four compounds are stable in powder and storable at room temperature. While S is commercially available, F, P, and I are synthesized in our laboratory (48, 49). When the CD dimers, P and I, were mixed with F in a 1:1:2 molar ratio, they formed the 1:1 inclusion complexes, hemoCD-P (F + P) and hemoCD-I (F + I), respectively, in the ferric iron(III) state. Small excess amounts of the CD dimer(s) (P or I, 1.1 equivalent to F) were added to prevent the presence of free F in the solution. We found that the solution containing the ferric complexes did not decompose under ambient conditions and is also storable at room temperature over one year (*SI Appendix*, Fig. S1). Solutions containing 3 to 14 mM of F were used in this study, which were readily reduced to iron(II) by adding excess S (5 mg/mL, 29 mM). The mixed solution of F, P, I, and S, i.e., the mixture of hemoCD-P and hemoCD-I in the iron(II) states, is hemoCD-Twins that is ready for injection to scavenge CO and CN^–^ simultaneously in vivo (Fig. 1 *C* and *D*). The molar concentration of F in the solutions is described as the concentration of hemoCDs.

**Fig. 2.**
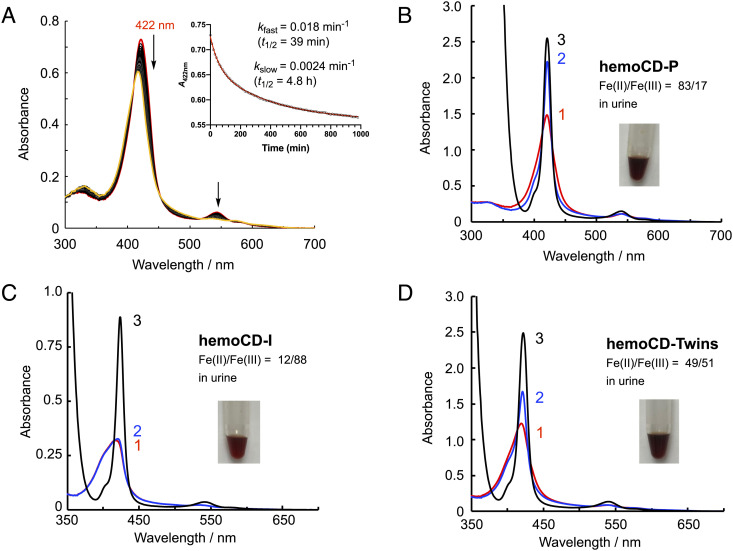
In vitro and in vivo characterization of hemoCD-Twins. (*A*) Autoxidation reaction of hemoCD-Twins (a mixture of ferrous hemoCD-P and hemoCD-I) in air-saturated PBS at 37 °C. The spectral changes indicated by the arrows reveal the autoxidation of the ferrous–O_2_ complexes to the ferric iron(III) states. The *Inset* shows a biphasic kinetic profile in the autoxidation reaction analyzed by double-exponential curve fitting to give first-order rate constants (*k*_fast_ and *k*_slow_). (*B*–*D*) Spectrophotometric analysis of diluted urine samples (see color in *Inset* pictures) collected from unanesthetized mice at 30 min after intraperitoneal (i.p.) injections (0.2 mL) of hemoCD-P (F = 7 mM, P = 8 mM, S = 29 mM) (*B*), hemoCD-I (F = 7 mM, I = 8 mM, S = 29 mM) (*C*), and hemoCD-Twins (F = 14 mM, P = 8 mM, I = 8 mM, S = 29 mM) (*D*). Absorption spectra were recorded before (1) and after successive additions of CO (2) and excess Na_2_S_2_O_4_ (3). From these spectra, the molar fractions of Fe(II) and Fe(III) states in the urine were determined as indicated in the respective panels.

When the solution of hemoCD-Twins (3.5 mM, 5 µL) was diluted in air-saturated PBS (3 mL), excess dithionite (S_2_O_4_^2–^) in the stock solution was rapidly consumed by O_2_ and dissolved in the buffer to be converted to sulfate (SO_4_^2–^) and H_2_O with superoxide and hydrogen peroxide as intermediates (50, 51). Due to residual O_2_ in the buffer, the O_2_ adducts of ferrous hemoCD-P and hemoCD-I were spontaneously formed in the diluted solution. The O_2_–ferrous complexes were gradually oxidized to their ferric iron(III) state, i.e., autoxidation (Fig. 2*A*). The absorbance decay was fitted by a double-exponential kinetic model, yielding *k*_fast_ = 0.018 min^–1^ and *k*_slow_ = 0.0024 min^–1^ at 37 °C. These parameters were the same as those independently measured and analyzed by a single-exponential kinetic model for hemoCD-P (*k*_autox_ = 0.0023 min^–1^, *t*_1/2_ = 5 h at 37 °C) and hemoCD-I (*k*_autox_ = 0.019 min^–1^, *t*_1/2_ = 36 min at 37 °C), respectively (*SI Appendix*, Fig. S2). Therefore, autoxidation of hemoCD-I is much faster than that of hemoCD-P, and the two compounds autoxidized independently even in their mixed system, i.e., hemoCD-Twins. The ferrous hemoCD-P bounds CO and ferric hemoCD-I bounds CN^–^ in solution. Indeed, hemoCD-Twins in air-saturated PBS showed dual reactivity to both CO and NaCN independent from their addition order (*SI Appendix*, Fig. S3). The ligand-binding affinities and kinetic parameters for hemoCD-P and hemoCD-I have been measured previously (39, 44, 46) (*SI Appendix,* Tables S1 and S2).

### Pharmacological Effects of hemoCD-Twins in Mice.

In this study, unanesthetized mice (20 ± 2 g, approximate circulating blood volume: 1.3 to 1.6 mL) (52) were used to test the safety and therapeutic effects of hemoCD-Twins against CO/CN^–^ intoxication. A PBS solution containing either hemoCD-Twins or its controls (hemoCD-P, hemoCD-I, or a vehicle control) were intraperitoneally (i.p.) administered to mice.

After injection (0.2 mL) of hemoCD-P (F = 7 mM, P = 8 mM), hemoCD-I (F = 7 mM, P = 8 mM), and hemoCD-Twins (F = 14 mM, P = 8 mM, I = 8 mM) with 5 mg/mL S (29 mM) in healthy conscious mice, brownish-red–colored urines were naturally excreted within 30 min (Fig. 2 *B*–*D*, *Inset*). The absorption spectra of the urine solutions confirmed the excretion of these compounds (Fig. 2 *B*–*D*). To investigate the oxidation state of the iron center, the urine solution (spectrum 1) was first charged with CO gas (spectrum 2) and then mixed with excess S (spectrum 3). In the case of hemoCD-P (Fig. 2*B*), spectrum 1 immediately changed to spectrum 2 with the sharp Soret at 422 nm typical of CO binding. In the case of hemoCD-I (Fig. 2*C*), little spectral changes were observed before (spectrum 1) or after addition of CO (spectrum 2). These results indicate that hemoCD-P was mostly excreted in its ferrous iron(II) form, while hemoCD-I was oxidized in the circulation and was then excreted in its ferric iron(III) form as confirmed in the spectra, showing that the compound is unable to react with CO. Based on the absorbances of these spectra (spectrum 1 to 3) at 422 nm, we calculated that the urine solutions contained 17% and 88% ferric iron(III) complexes of hemoCD-P and hemoCD-I, respectively. Compared to the in vitro characterization, autoxidation of hemoCD-I (*t*_1/2_ = 36 to 39 min in vitro) might be accelerated in vivo (88% autoxidized in 30 min), probably due to difference in the two biological milieux. The autoxidation rate of the oxy–iron(II) complexes in H_2_O is significantly influenced by coexisting anions as demonstrated previously by Shikama (53) and by our group (38). Consistent with the results obtained after injections of hemoCD-P and hemoCD-I, the urine contained around half of the ferric iron(III) complex (51%) after injection of hemoCD-Twins. These results indicate that, once hemoCD-Twins is injected, hemoCD-P circulates by mostly maintaining its ferrous iron(II) state that can capture CO, while ferrous hemoCD-I is oxidized to its ferric iron(III) complex that is ready for binding to CN^–^. Therefore, hemoCD-Twins functions as a dual scavenger of CO and CN^–^ in vivo.

We also found that 24 h after treatment with hemoCD-Twins, all the mice showed no difference in behavior compared to controls and no residual porphyrin compound was visually observed in major organs (brain, lung, heart, liver, spleen, and kidney) (*SI Appendix*, Fig. S4). Histopathological data showed no significant abnormalities in these tissues after administration of hemoCD-Twins (*SI Appendix*, Fig. S5). Moreover, there were no significant changes in biochemical markers of kidney and liver functions [blood urea nitrogen (BUN), creatinine (CRE), alanine aminotransferase (ALT), aspartate aminotransferase (AST), and lactate dehydrogenase (LDH)] compared to controls treated with PBS (*SI Appendix*, Fig. S6). These results confirm the safety of hemoCD-Twins in mice. To exclude a possible toxicity of the reducing agent sodium dithionite (S), mice were challenged with this compound at various doses administered i.p. (*SI Appendix*, Table S3). The dose of 1,000 mg/kg S was found to be lethal. The dose of S present in the complex mixture of hemoCD-Twins (5 mg/mL in 0.2 mL per mouse of 20 ± 2 g) calculated at 50 mg/kg is 20 times lower than the toxic dose of S alone observed in mice.

### hemoCD-Twins Is an Effective Antidote against CO and CN^–^ Poisoning in Mice.

**Fig. 3.**
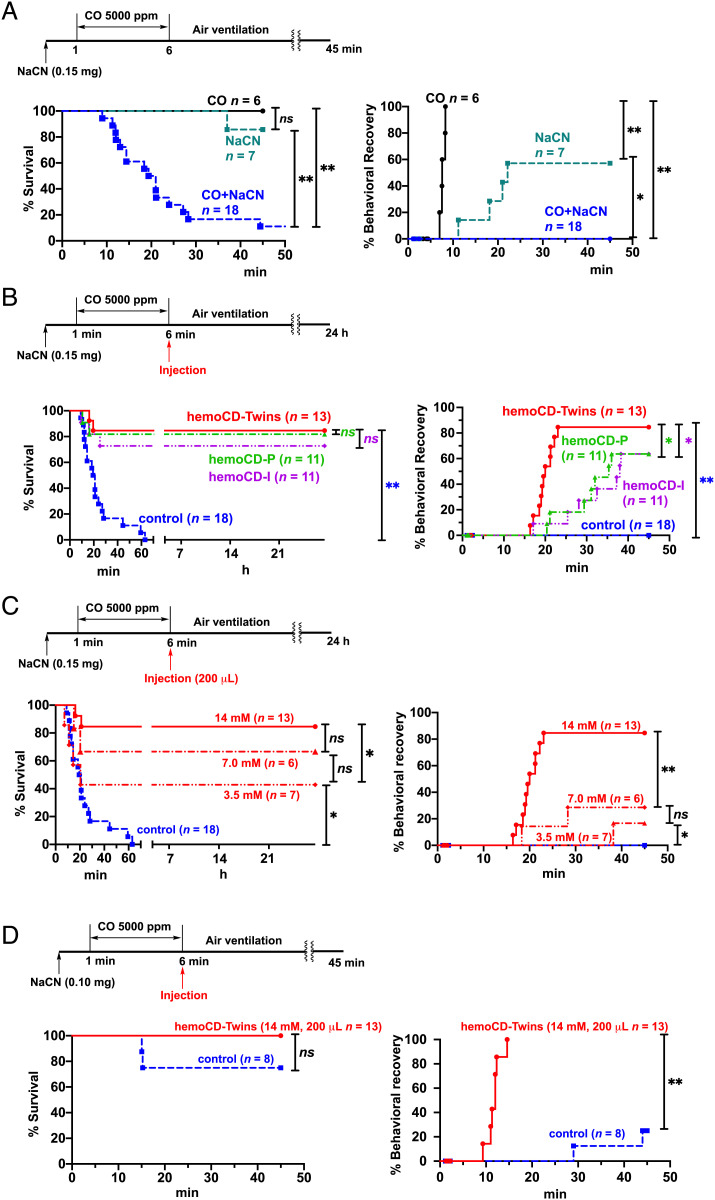
hemoCD-Twins as an effective antidote against CO and CN^–^ mixed poisoning in mice. The toxic effect of CO and CN^–^ was evaluated by assessing the survival rate (*Left*) and behavioral recovery of mice from their incapacitation (*Right*). (*A*) Toxic effects of NaCN (oral administration in PBS), CO gas exposure (5,000 ppm in air), or a combination of the two treatments in mice. (*B*) Effect of intraperitoneal injection of hemoCD-Twins (F = 14 mM, P = I = 8 mM with 5 mg/mL of S) on mice survival rate and mice behavioral recovery. hemoCD-P (F = 7 mM, P = 8 mM) or hemoCD-I (F = 7 mM, I = 8 mM) with 5 mg/mL of S were used as controls for comparison. (*C*) Dose-dependent effects of hemoCD-Twins in mice after exposure to the CO/CN^–^ mixed intoxication. (*D*) Effect of hemoCD-Twins (14 mM, 0.2 mL) on nonlethal doses of CO and CN^–^ mixed intoxication (NaCN: 0.1 mg, CO: 5,000 ppm). Statistical significance: *P < 0.05, **P < 0.01; *ns*, not significant.

In the next set of experiments, we assessed the potential of using hemoCD-Twins as an antidote against gas poisoning. First, the toxicity of CO and CN^–^ was tested in mice. The survival curves after exposure of mice to controlled CO atmospheres of 5,000, 7,500, and 10,000 ppm for 5 min showed a dose-dependent effect (*SI Appendix*, Fig. S7*A*). While 10,000 ppm CO caused significant lethality (86%), 7,500 and 5,000 ppm CO were less toxic, inducing 33 and 0% lethality, respectively. Indeed, these two lower doses were associated with a gradual recovery of mice from their physical incapacitation (see behavioral recovery, *SI Appendix*, Fig. S7*B*). Similarly, the effects of oral administration of NaCN (0.10, 0.15, and 0.20 mg per 20 ± 2 g mice) dissolved in PBS (0.1 mL) were also tested (*SI Appendix*, Fig. S7 *C* and *D*), and a significant lethality was found at 0.20 mg NaCN. Notably, while most mice survived when independently challenged with either 0.15 mg NaCN or 5,000 ppm CO, a rapid lethal toxicity was observed within 40 min when these two compounds were administered simultaneously (Fig. 3*A*). Strikingly, injection of mice with hemoCD-Twins significantly reduced lethality caused by the combined exposure to CO and CN^–^ (Fig. 3 *B*, *Left*), resulting in approximately 85% of mice surviving 24 h after treatment (*n* = 11/13). Blood carboxyhemoglobin (CO-Hb) and CN^–^ levels in mice reached a maximum of 51% and 2.1 µg/mL, respectively. These values decreased markedly to 8.8% and 0.31 µg/mL, respectively, 45 min after administration of hemoCD-Twins (*SI Appendix*, Fig. S8). In addition, hemoCD-Twins-treated mice recovered from their physical incapacitation within 15 min after injection (Fig. 3 *B*, *Right*); that is, mice that became unconscious due to the simultaneous poisoning by CO and CN^–^ regained their mobility and began to walk within a short time after administration of hemoCD-Twins. We note that treatment with either hemoCD-P or hemoCD-I after combined exposure of mice to CO and NaCN also showed significant effects in reducing lethality and enhancing the behavioral recovery, although the recovery rates were slower and less pronounced than those of mice receiving the dual antidote system, hemoCD-Twins (Fig. 3*B*).

A better recovery after hemoCD-Twins treatment was also evident when looking at the lactate levels in blood (*SI Appendix*, Fig. S9). In fact, lactate concentration increased from 5.9 ± 1.5 mM to 19.2 ± 3.1 mM at 6 min after combined exposure to CO and CN^–^ and significantly decreased to 6.5 ± 1.1, 12.3 ± 1.1, and 16.8 ± 4.1 mM at 39 min (*t*_45_) after injections of hemoCD-Twins, hemoCD-P, and hemoCD-I, respectively. Therefore, hemoCD-Twins exerted a marked therapeutic effect on lactic acidosis induced by CO and CN^–^ (30, 54–56). Of note is that the survival rate and behavioral recovery upon injection of hemoCD-Twins increased in a dose-dependent manner (Fig. 3*C*), with 14 mM hemoCD-Twins showing the best prosurvival effect. A rapid recovery from incapacitation was also demonstrated in the nonlethal mice model (0.10 mg NaCN then 5,000 ppm CO for 5 min) (Fig. 3*D*). The fractions of the complexes bound to CO and CN^–^ and found in the urine were also dependent on the doses of the complexes administered (*SI Appendix*, Figs. S10 and S11). When hemoCD-P or hemoCD-I at the doses of 1.0, 3.5, or 7.0 mM was injected into mice intoxicated with 5,000 ppm CO or 0.1 mg NaCN, these compounds were excreted in urine bound to their respective ligands with different degrees of saturation. While at the lower doses the complexes were almost saturated by these ligands, we measured 53% saturation of hemoCD-P with CO and 67% saturation of hemoCD-I with CN^–^ at the highest dose used. Thus, by injecting a dose of 7 mM, there is still enough compound in the circulation with the capacity to bind additional CO or CN^–^. These results demonstrate that the dose of 7 mM hemoCD-P or hemoCD-I (i.e., 14 mM of hemoCD-Twins) is sufficient to treat mice and neutralize the CO/CN^–^ mixed poisoning used in our protocol.

### Improved Survival Rate and Behavioral Recovery in Mice Treated with hemoCD-Twins Following Exposure to Lethal Combustion Gases.

**Fig. 4.**
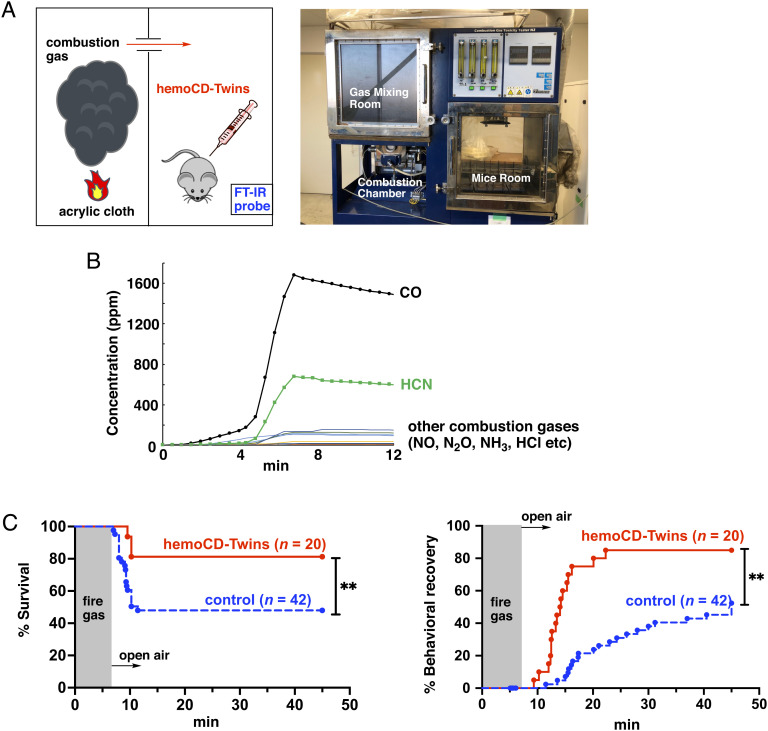
Antidotal action of hemoCD-Twins in mice exposed to gas from burning acrylic cloth. (*A*) Representation of the experimental setup. The detailed configuration of this device is described in *SI Appendix*. (*B*) Time-course generation of the major components present in the gas mixture during combustion of acrylic cloth (17 g, 220 mm × 220 mm). The gas components were quantitatively measured using a real-time FT-IR probe connected to the mice chamber. (*C*) Survival rate and behavioral recovery profiles of mice exposed to the gas combustion ± hemoCD-Twins after incapacitation (6.5 to 7.0 min exposure to the combustion gas). Statistical significance, **P < 0.01.

To further test the therapeutic effects of hemoCD-Twins in a real-life scenario, combustion gas from acrylic cloth was used to poison mice in a combustion gas toxicity tester (Fig. 4*A* and *SI Appendix*, Fig. S12). During the combustion phase, the gas components in the mice chamber were quantitatively analyzed by a Fourier transform infrared spectroscopy (FT-IR) (57), revealing significant amounts of CO and HCN being simultaneously generated (Fig. 4*B*). The gas concentrations reached a plateau at 5 min. Approximately 1,600 ppm CO and 700 ppm HCN were generated during combustion of *ca* 17 g of acrylic cloth, while other harmful gases such as NO, N_2_O, and NH_3_ were produced in much smaller amount. Mice were exposed to the combustion gas and were physically incapacitated after 6 to 7 min. The mice were then removed from the chamber and hemoCD-Twins (F = 14 mM, P = I = 8 mM, 0.2 mL with 5 mg/mL of S) was administered. As shown in Fig. 4*C*, the antidotal action of hemoCD-Twins on survival rate and behavioral recovery was significant as compared to untreated controls. In this experiment, only mice that were still breathing when removed from the chamber box were used for hemoCD-Twins treatment. Approximately 10% of the animals died during gas exposure and these mice were excluded from data collection.

### Pharmacological Effects of hemoCD-Twins in Rats.

Male rats (300 ± 15 g, approximate circulating blood volume: 18 to 22 mL) were anesthetized with isoflurane and used to assess the pharmacokinetic of hemoCD-Twins and its effect on hemodynamic parameters. The toxicological effects of CO and HCN in this model are assumed to be essentially the same as the ones produced in mice, with no particular relevance about differences in species or sex.

**Fig. 5.**
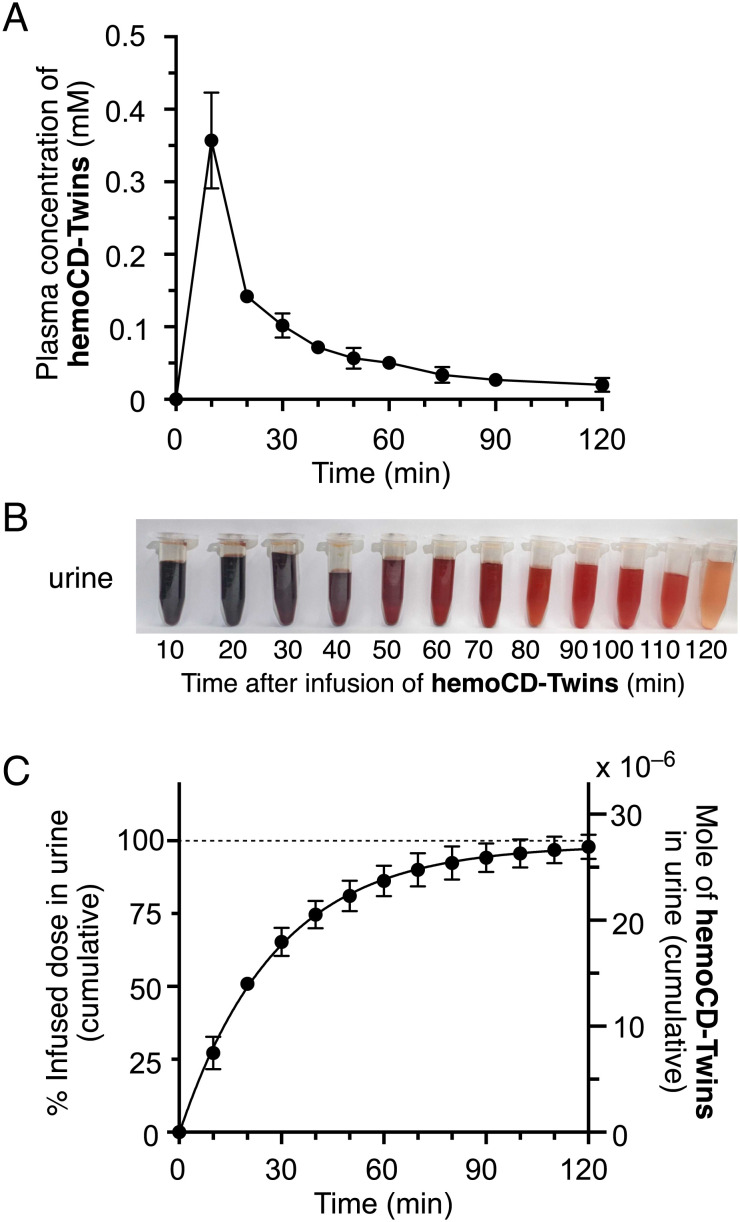
Pharmacokinetic profile of hemoCD-Twins after intravenous infusion in rats. A solution of hemoCD-Twins (2 mL, 14 mM) was infused through the femoral vein of anesthetized rats at a rate of 12 mL/h for 10 min. Blood and urine samples were collected during and after infusion of hemoCD-Twins as a function of time. (*A*) Plasma clearance profile. (*B*) A photograph of urine samples collected at different time points after infusion of hemoCD-Twins. (*C*) Urinary excretion profile. Each plot represents the mean ± SD (*n* = 3 to 6).

A PBS solution (2 mL) of hemoCD-Twins (14 mM) or its controls (hemoCD-P, hemoCD-I, or a vehicle control) was intravenously (i.v.) infused into the femoral vein of anesthetized rats. After starting the infusion of 2 mL of hemoCD-Twins at the rate of 12 mL/h (total duration ~ 10 min), blood plasma samples were collected at different time points and the amount of hemoCD-Twins was quantified by size exclusion chromatography (*SI Appendix*, Fig. S13). The plasma concentration of hemoCD-Twins increased immediately and then rapidly decreased after infusion (Fig. 5*A*). Based on this profile, renal clearance rate, elimination half-life time, and area under the curve of the hemoCD-Twins concentration in plasma over time were 26.5 ± 4.8 mL/min, 47.1 ± 17.3 min, and 1.1 ± 0.2 µmol min/mL, respectively. The urinary excretion was quantified by UV-vis spectroscopic measurement, showing that *ca.* 98% of the injected hemoCD-Twins was excreted within 2 h (Fig. 5 *B* and *C*). The rapid renal clearance is ascribed to the relatively small molecular size (*ca.* 2.8 nm) of the complex, its hydrophilicity, and little interaction with plasma proteins as we have previously determined when studying the pharmacokinetics of hemoCD-P (41). During the excretion of hemoCD-Twins, the ferric and ferrous complexes in urines were found at an approximately 1:1 molar ratio (*SI Appendix*, Fig. S14), indicating that the infused hemoCD-P and hemoCD-I were eliminated via renal clearance at similar rates. Rapid infusion (12 mL/h) of hemoCD-Twins to healthy rats showed no change in their heart rate and a slight increase in mean blood pressure (*SI Appendix*, Fig. S15). Biochemical markers (BUN, CRE, LDH, ALT, and AST) indicative of renal and liver functions assessed in rat plasma before and 3 h after infusion (*SI Appendix*, Fig. S16) revealed small effects of hemoCD-Twins on these parameters except for BUN. The increase in BUN (from 24.0 to 38.6 mg/dL) is probably due to the presence of some residual hemoCD-Twins still present in the body 3 h after administration.

**Fig. 6.**
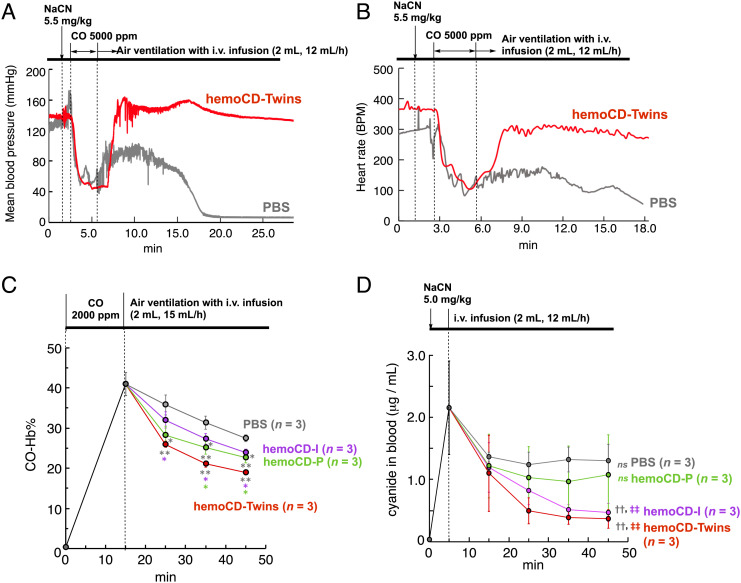
Hemodynamic parameters and blood CO/CN^−^ levels in rats before and after infusion of hemoCD-Twins. (*A* and *B*) Time course of mean blood pressure (*A*) and heart rate (*B*) in rats after successive challenge with NaCN (5.5 mg/kg in PBS, oral administration), CO gas (5,000 ppm inhalation), and i.v. infusion of hemoCD-Twins (14 mM, 2 mL in PBS, infused at the rate of 12 mL/min) or PBS under air ventilation. Data from single animals are displayed due to the instability of these parameters following exposure to CN^–^ and CO. Data from additional animals are shown in *SI Appendix*, Fig. S17. (*C* and *D*) Effect of hemoCD-Twins on blood CO-Hb% (*C*) and CN^–^ concentration (*D*) after exposure to CO (2,000 ppm inhalation) or oral administration of NaCN (5.0 mg/kg), respectively. In these nonlethal models, hemoCD-Twins (14 mM, 2 mL) was infused using a syringe pump at the rate of 15 mL/h (*C*) or 12 mL/h (*D*). hemoCD-P (7 mM, 2 mL), hemoCD-I (7 mM, 2 mL), and PBS (2 mL) were used as controls. Statistical significance: *P < 0.05, **P < 0.01, compared to other curves at each time point; ^††^*P* < 0.01, compared to PBS treatment; ^‡‡^*P* < 0.01, compared with the 5 and 45 min time points for each group; *ns*, not significant.

Successive additions of NaCN (5.5 mg/kg, oral administration) and CO gas (5,000 ppm, 3 min) to the anesthetized rats significantly reduced mean arterial pressure and heart rate as shown in Fig. 6 *A* and *B*, respectively. Interestingly, i.v. infusion of hemoCD-Twins following exposure to CO/NaCN resulted in a more pronounced restoration of blood pressure and heart rate compared to rats infused with PBS (control). To demonstrate that hemoCD-Twins effectively neutralizes CO and CN^–^, the rate profiles for CO-Hb% and CN^–^ concentrations in blood were measured in their respective nonlethal CO/CN^–^ models. hemoCD-Twins induced a marked reduction of blood CO-Hb% and CN^–^ levels compared to PBS controls after exposure of rats to either CO or NaCN (Fig. 6 *C* and *D*). hemoCD-P and hemoCD-I also caused a significant but less pronounced reduction in CO-Hb% compared to hemoCD-Twins. As expected from the reported gas-binding parameters (*SI Appendix,* Table S1), hemoCD-P elicited a more rapid decrease in CO-Hb% than hemoCD-I. In contrast, hemoCD-I markedly reduced CN^–^ levels (Fig. 6*D*) while hemoCD-P was much less effective. These data clearly indicate that: 1) hemoCD-Twins exerts a dual therapeutic effect to counteract simultaneous CO and CN^–^ poisoning and 2) the antidote activity of hemoCD-Twins is superior to that of hemoCD-P and hemoCD-I when used alone.

## Discussion

In the case of fire accidents and intoxication by combustion gas in humans, a storable, ready-to-use, quick-acting antidote would be highly desirable for in-situ treatment. In this study, we report on the invention of such antidote substance, hemoCD-Twins, using biomimetic heme protein model complexes. The components of hemoCD-Twins are stable for over one year with no sign of decomposition when stored in the ferric iron(III) solution. When injected, ferrous hemoCD-P captures CO, while ferrous hemoCD-I is readily oxidized to its ferric iron(III) form and captures CN^–^. In addition, the compound is readily excreted in urine and totally disappears from the body within few hours. Using various experimental models, we show that hemoCD-Twins significantly protects mice and rats from poisoning and death caused by the combination of CO and CN^–^ exposure or by combustion gas produced during the burning of acrylic cloth. Thus, hemoCD-Twins is a stable and urinary-excretable compound that functions as an antidote against CO and CN^–^ in vivo.

As it is well recognized, CO poisoning frequently occurs in fire accidents. Generally, victims who die because of CO poisoning tend to exhibit over 60% CO-Hb in their blood. On the contrary, death in subjects with less than 30% CO-Hb has been also frequently reported (58, 59). One explanation for this variability could be the simultaneous poisoning of CO and HCN. As we demonstrated in this study, combustion of acrylic cloth simultaneously produced significant amounts of CO (1,600 ppm) and HCN (700 ppm) gases. It should be noted that HCN is generated at lower concentration than CO, but its toxic effect is 20 to 40 times stronger than that of CO (7, 26–27, 28). Therefore, the combined inhalation of CO and HCN mixed gas may synergize to cause severe toxic effects of these gases even in concentrations that are much lower than their own inherent toxic level (26, 30, 55, 56, 60). This possibility is supported by our data showing that exposures of animals to either 5,000 ppm CO gas or 0.15 mg NaCN alone were not lethal, but the two compounds administered simultaneously induced very high mortality. Therefore, simultaneous detoxification of CO and CN^–^ should have high relevance in the context of fire gas poisoning, but this issue has not been addressed experimentally.

It is important to note that treatments against CO poisoning have been developed independently from CN^–^ antidotes. For CO poisoning, normobaric/hyperbaric O_2_ ventilations are practically applied; this intervention requires transportation of patients to hospitals equipped with O_2_ cylinders and normobaric/hyperbaric chambers, causing significant time delays (32, 33, 58, 61). To overcome this limitation, an injectable CO antidote has been recently proposed using a neuroglobin mutant (34). The mutant (hNgb_H64Q-CCC_) showed much higher CO binding affinity than Hb in red blood cells and thus rapidly decreased CO-Hb levels and improved survival rate when administered to mice exposed to CO. The functional mechanism of hemoCD-P as a CO antidote is basically the same as that of the neuroglobin mutant. The high CO-binding affinity and the CO/O_2_ selectively of hemoCD-P make this complex able to remove CO from native heme proteins with high efficiency (*SI Appendix*, Table S1). Moreover, the compound shows a slow autoxidation of the iron(II) complexes, satisfying the requirement for an in vivo CO scavenging molecule. Accordingly, our group has recently shown that an intravenous infusion of hemoCD-P to rats exposed to toxic CO levels promptly reduces CO bound to Hb and accumulation of CO in organs and tissues including the brain (44).

As for CN^–^ poisoning, ferric met-Hb and hydroxocobalamin (OHCbl) are currently adopted as therapeutic interventions (11). Met-Hb can be endogenously formed by injecting nitrite compounds (amyl nitrite and/or sodium nitrite) as the oxidants, and its ferric heme will capture CN^–^ (*SI Appendix*, Table S2). However, treatment with nitrite compounds causes significant disruption of O_2_ transport by ferrous Hb in red blood cells and thus it is not an ideal compound to be used in the case of fire accidents where CO intoxication is possibly concurrent (37). OHCbl is used as an injectable CN^–^ scavenger (11, 36, 62); the cobalt(III) center of OHCbl has a high binding affinity for CN^–^, which binds through ligand exchange with the hydroxide ion of OHCbl. However, one downside is that OHCbl strongly binds to plasma proteins, slowing down its clearance (it takes over 30 d for elimination) (63). Additionally, interaction with serum albumin inhibits the binding of CN^–^ to the cobalt(III) center of OHCbl (46). Compared to these systems, hemoCD-I is much more advantageous as a CN^–^ scavenger in vivo because it is less affected by serum proteins, keeping its high CN^–^-binding ability even in the presence of serum albumin (46).

hemoCD-Twins is the example of an injectable antidote that, after a single administration, is effective against simultaneous CO and CN^–^ poisoning. The antidotal effect of hemoCD-Twins on CO and CN^–^ mixed poisoning was remarkable in the mice and rat models examined in this study. We used conscious mice for survival tests using a gas-tight chamber (4 L) and injected them intraperitoneally with hemoCD-Twins for quick intervention. In addition, we used anesthetized rats for pharmacokinetic and hemodynamic studies because of the ease of surgery and intravenous administration of the compounds. We observed that the toxic effects of CO and CN^–^ are basically the same in mice and rats; therefore, the results obtained from these two rodent species are discussed here without any distinction. Because of a high transfer efficiency from the abdominal cavity to blood (>50% for hemoCD-P) (42), both i.p. and i.v. injections resulted in a similar antidotal effect. Importantly, the therapeutic effect of hemoCD-Twins was recapitulated in a real-life simulation of fire gas intoxication, demonstrating that the survival rate and behavioral recovery of animals breathing gas from burning acrylic cloth were markedly increased by treatment with the dual antidote. Considering the possible translation of this approach in humans, approximately 200 to 400 mL of hemoCD-Twins (14 mM) solution would be needed for one treatment, which would be ideally infused i.v. To prepare a solution of 400 mL hemoCD-Twins, approximately 6 g F, 9 g P and I, and 2 g S should be dissolved in saline. These quantities are readily achievable because the synthetic procedures have been established in gram scales (46, 48). Indeed, we usually synthesize 2 to 4 g of the CD dimers (P and I) at once in the laboratory while F and S are commercially available.

Concerning the safety of new compounds, we have repeatedly assessed that both hemoCD-P and hemoCD-I show little cytotoxicity, hemolytic activity, and marginal effects on hemodynamic parameters upon their administration to mice and rats (41, 44, 46, 47, 64). In our protocols, sodium dithionite (S) was coinjected as this compound is needed to keep **hemoCD**s in its reduced state. We have previously used hemoCD-P in vivo after removing excess S by passing the reduced complex through a desalting column (42, 44, 65). However, this purification process is time-consuming and impractical for use in emergency situations. Thus, in the present study, we omitted this step to support the potential translation to the clinic of our findings. In this context, we point out that the reported toxicity of S is low (LD_50_ = 2,500 mg/kg for rats, oral administration) (66) comparable to the one reported for NaCl (LD_50_ = 3,000 mg/kg for rats, oral administration), and we confirmed in our experiments a lethal effect of S at 1,000 mg/kg following i.p. injection in mice (*SI Appendix*, Table S3). The amount of S contained in hemoCD-Twins is 50 mg/kg (29 mM), far below the reported toxic levels. In addition, approximately half a mole of dithionite (14 mM) is consumed to reduce hemoCD-Twins before injection. Therefore, the actual dose of dithionite administered would be below 25 mg/kg, which is much lower than the toxic one. Indeed, hemodynamic, histopathological, and biochemical analyses of healthy rodents treated with hemoCD-Twins at the effective doses (14 mM, 10 to 13% volume to circulating blood) showed negligible effects. Among the additional reducing agents we have tested including ascorbate, sulfite, metabisulfite, and methylene blue, dithionite (S) appears to be the most efficient one to rapidly and quantitatively form the ferrous iron(II)porphyrin complexes. We understand that further safety tests and optimization of our antidote formulation would be necessary for clinical use of S as a reducing agent, but the data presented here appear promising. Another important issue to consider is that once in the circulation, hemoCD-I in the reduced state can be readily autoxidized and this may lead to the production of superoxide anion (O_2_ + Fe(II) → Fe(III) + O_2_^•–^). The influence of superoxide and other related reactive O_2_ species has not been investigated here, but the presence of small amount of superoxide dismutase and catalase as adjuvants to the hemoCD-Twins formulation might be considered in future studies.

It is interesting to note that CO intoxication can be quickly diagnosed by blood gas analyzers (e.g., pulse CO oximetry), while measuring CN^–^ concentrations in blood is quite time-consuming (as we experienced in rat blood tests), perhaps explaining why CN^–^ intoxication has been often ignored in the diagnosis. Indeed, in forensic anatomy of fire-related accidents from 2007 to 2009 in Japan (67), measurements of CO-Hb was carried out in 98.3% of cases, while tests to determine CN^–^ concentration were performed in only 13% of cases. In most cases, fire accidents result in high levels of CO, whereas HCN is not always produced (e.g., wildfires or vehicle exhaust). Mixed intoxication of CO and HCN is a major and often-overlooked factor in the case of building/urban fires. We envisage that hemoCD-Twins can be quickly administered to patients suffering from fire gas exposure without the need to wait for the diagnosis of whether patients are primarily intoxicated by CO and/or HCN. Additionally, hemoCD-Twins has the potential to act as an antidote to other toxic gases such as nitric oxide and hydrogen sulfide that can also bind to heme-related proteins, which are currently being investigated in our laboratory (68, 69). Finally, we emphasize that hemoCD-Twins reversed the increase in lactic acidosis typical of CO and CN^–^ intoxication, strongly suggesting that scavenging of these two poisonous gases by our antidote improves tissue oxygenation.

In conclusion, we have developed hemoCD-Twins as an injectable and urinary-excretable antidote against CO and CN^–^ mixed poisoning. The biochemical and preclinical studies presented here indicate promising therapeutic features of this compound based on its redox properties, pharmacokinetic behavior, minimal side effects, and effective scavenging activities against CO and CN^–^. Future tests in large mammals and humans will provide definite and conclusive evidence for the efficacy of hemoCD-Twins as a breakthrough antidote that might save human lives from frequently occurring gas poisoning episodes worldwide.

## Materials and Methods

### Materials.

Fe^III^TPPS (F), Py3CD (P), and Im3CD (I) were synthesized in our laboratory. The synthetic procedures were previously reported (46, 48, 70). Sodium dithionite (S), sodium cyanide (NaCN), phosphate-buffered saline (PBS), and other chemicals were purchased from Fujifilm Wako.

### Preparation and Characterization of hemoCD-Twins.

To prepare the solution of hemoCD-Twins, three solid compounds, F, P, and I, were first dissolved in PBS to give the stable ferric iron(III) solution. Before injection to animals, the ferric iron(III) solution was reduced by mixing with 5 mg/mL S to give the ferrous iron(II) solution. Autoxidation reaction from the iron(II) to iron(III) complexes was monitored by UV-vis spectroscopy (Shimadzu UV-2450 spectrophotometer). Detailed procedures of sample preparation and chemical characterization are available in *SI Appendix*.

### Animal Experiments.

All animal studies were performed under the approval of Doshisha University, Doshisha Women’s College of Liberal Arts, Tokai University, Building Research Institute, and Center for Better Living. For monitoring the therapeutic effect of hemoCD-Twins on lethal CO/CN^–^ intoxication models including the model using combustion gases, we used female BALB/cCrSlc mice. After the mice were exposed to these toxins, the solution of hemoCD-Twins in PBS (0.2 mL) was injected intraperitoneally. The mice were then monitored for survival rate and behavioral recovery under aerobic conditions at room temperature. For pharmacokinetic and hemodynamic studies, we used male Wistar rats (300 ± 15 g). Rats anesthetized with isoflurane were infused i.v. with hemoCD-Twins. During and after the infusion, heart rate and blood pressure were continuously monitored using Bio Amps (AD Instruments Ltd). Plasma and urine concentrations of hemoCD-Twins in rats were quantified by UV-vis spectroscopy (Shimadzu UV-2450 spectrophotometer) and size exclusion chromatography (NGC Chromatography System, Bio-Rad Laboratories Inc, attached with an ENrich SEC650 10 × 300 column), respectively. A full description of these animal experiments is available in *SI Appendix*.

### Statistical Analysis.

Statistical analyses were performed using GraphPad Prism, Version 8.0 (GraphPad Software). All data represent the means ± SE from at least three different experiments and were analyzed by Student’s *t* test. Survival curves were analyzed using Kaplan–Meier curves and log-rank test. Differences with *P* values of less than 0.05 were considered significant.

## Supplementary Material

Appendix 01 (PDF)

## Data Availability

All study data are included in the article and/or *SI Appendix*.
