# Supplementary material for: A synthetic porphyrin as an effective dual antidote against carbon monoxide and cyanide poisoning
Source: Proc Natl Acad Sci U S A. 2023 Feb 21;120(9):e2209924120. doi: 10.1073/pnas.2209924120 (PMC9992825; doi:10.1073/pnas.2209924120)
Supplement: Supplementary file 1 — Appendix 01 (PDF) [file pnas.2209924120.sapp.pdf]

Supplementary Information for

A Synthetic Porphyrin as an Effective Dual Antidote against Carbon Monoxide and Cyanide Poisoning

Qiyue Mao, Xuansu Zhao, Akiko Kiriyama, Shigeru Negi, Yasutaka Fukuda, Hideki Yoshioka, Akira T. Kawaguchi, Roberto Motterlini, Roberta Foresti, Hiroaki Kitagishi*

*Corresponding author: Hiroaki Kitagishi
Email: hkitagis@mail.doshisha.ac.jp

This PDF file includes:

Abbreviation list
Materials and Methods
SI References
Tables S1 to S3
Figures S1 to S17

Abbreviation List:

FeTPPS or **F**	5,10,15,20-Tetrakis(4-sulfonatophenyl)porphinatoiron(II/III)
CD	cyclodextrin
Py3CD or **P**	Per-*O*-methylated β-cyclodextrin dimer linked by a pyridine ligand at the 3,3'-positions of two glucopyranose units
Im3CD or **I**	Per-*O*-methylated β-cyclodextrin dimer linked by an imidazole ligand at the 3,3'-positions two glucopyranose units
**S**	Sodium dithionite
**hemoCD-P**	A 1:1 inclusion complex of **F** with **P**
**hemoCD-I**	A 1:1 inclusion complex of **F** with **I**
**hemoCD-Twins**	An equimolar mixture of **hemoCD-P** and **hemoCD-I**
PBS	Phosphate buffered saline
Hb	Hemoglobin
Mb	Myoglobin
CO-Hb	Carboxyhemoglobin
OHCbl	Hydroxocobalamin
Ngb	Neuroglobin
C*c*O	Cytochrome *c* oxidase
i.p.	Intraperitoneal or intraperitoneally
i.v.	Intravenous or intravenously
BUN	Blood urea nitrogen
CRE	Creatinine
ALT	Alanine aminotransferase
AST	Aspartate aminotransferase
LDH	Lactate dehydrogenase
FT-IR	Fourier transform infrared spectroscopy

MATERIALS AND METHODS

Study design. The objective of this study is to investigate the efficacy of simultaneous removal of CO and cyanide from the intoxicated animals using a synthetic porphyrin (**F**) mixed with two cyclodextrin dimers (**P** and **I**) and a reducing agent (**S**). We describe herein the preparation of an antidote solution named **hemoCD-Twins**, consisting of **F**, **P**, **I**, and **S** in PBS. The basic chemical properties of **hemoCD-Twins** were characterized by UV-vis spectrometry. All animal studies were performed under the approval of Doshisha University, Doshisha Women's College of Liberal Arts, Tokai University, Building Research Institute, and Center for Better Living. We used two types of rodents (mice and rats) in this study. The toxic effects of CO and HCN are assumed to be fundamentally the same in rats and mice, and any difference due to the species and sex used were not specifically examined in this study. The pharmacokinetic and safety profiles of **hemoCD-Twins** using mice and rats were performed at Doshisha University and Doshisha Women's College of Liberal Arts. The experiments using a mouse model of the CO and cyanide mixed intoxication and the efficacy of **hemoCD-Twins** as CO and cyanide antidote were conducted at Doshisha University. Hemodynamic parameters and blood CO and cyanide concentrations were assessed at Doshisha Women's College of Liberal Arts and Tokai University using a model of CO/cyanide non-lethal intoxication in rats. Finally, we used a combustion gas to simulate real-life fire accidents and evaluated the antidotal effect of **hemoCD-Twins** in mice against combustion gas using an experimental set up at the Building Research Institute and Center for Better Living. In the lethal mouse model, data from the animals that died during the exposure in the chamber were excluded from the evaluation tests, while all other data were included. With respect to selection bias, group of mice and rats at similar body weight were used for all experiments, and treatment with either **hemoCD-Twins** or control compounds were selected completely randomly and administered repeatedly by different experimenters. Administration of the compounds in a blind manner was not possible because the specific coloration of the solutions used would have given indication of their content to the investigator.

Materials. Fe^{III}TPPS (**F**), Py3CD (**P**), and Im3CD (**I**) were synthesized in our laboratory. The synthetic procedures were the same as previously reported (1–3). Sodium dithionite (**S**), sodium cyanide (NaCN), and phosphate buffered saline (PBS), and other chemicals were purchased from Fujifilm Wako.

Preparation of hemoCD-Twins. To prepare the solution of **hemoCD-Twins**, three powder compounds (**F**, **P**, and **I**) were used at a molar raito **F**:**P**:**I** = 2:1:1. For example, **F** (16 mg, MW = 1148 Da as the pentahydrate) (3), **P** (24 mg, MW = 3 kDa), and **I** (24 mg, MW = 3 kDa) were dissolved in PBS (1 mL) using a vortex mixer to give the ferric iron(III) stock solution (14 mM based on **F**). A small excess of **P** and **I** (1.1 equiv) was added to prevent the presence of free **F** in solution. The solution was then mixed well with **S** (5 mg/mL, 29 mM) to give the ferrous iron(II) solution (**hemoCD-Twins**). The solution of **hemoCD-Twins** at **F** = 14 mM was typically used for administration *in vivo*. To prepare the solutions of **hemoCD-P** and **hemoCD-I**, the respective compounds were weighed at **F**:**P** = **F**:**I** = 1.0:1.1 molar ratio (*i.e.*, **F** = 7 mM) with **S** = 5 mg/mL at fixed concentrations.

Autoxidation measurements. Stock solutions of **hemoCD-P**, **hemoCD-I**, and **hemoCD-Twins** (1–5 μL) were appropriately diluted into air saturated PBS (3 mL). Upon dilution, residual **S** in the stock solution was readily decomposed by O_2, and this was confirmed by the absence of the absorption band at around 320 nm due to $S_2O_4^{2-}$. The O_2 adducts of ferrous **hemoCD**s (**hemoCD-P**, **hemoCD-I**, and the mixture) were spontaneously formed with residual O_2 in the air-saturated buffer solution. The spectral characterization of the O_2 complexes were reported previously (4–6). Autoxidation reaction from the iron(II) to iron(III) complex was monitored by repeated scans of the UV-vis spectrum (Shimadzu UV-2450 spectrophotometer, 300–700 nm), where the absorbance changes were plotted as a function of time and analyzed by a single or double exponential curve fitting using GraphPad Prism, Version 8.0 (GraphPad Software) to determine the rate constants.

Urinary analysis in mice. Studies on animals were carried out under the approval of the Guidelines for Animal Experiments of Doshisha University. Animals were first acclimatized for one week at 26 ± 0.5°C with access to water and food ad libitum. The solution of **hemoCD-P**, **hemoCD-I**, and **hemoCD-Twins** (7 or 14 mM, 200 μL in PBS) were injected intraperitoneally to female BALB/cCrSlc mice (9–11 weeks; mean weight: 20 g). After injection, the urine excreted was collected for one hour. The color of the urine was brownish-red due to excretion of the **hemoCD** components. Urine samples were appropriately diluted for UV-vis measurements. The ratio of the iron(II) and iron(III) complex in urine was confirmed by successive additions of CO and **S** to the solution. The calculations using the respective molar absorption coefficients (met-, oxy-, and CO-complexes) of **hemoCD-P** and **hemoCD-I** were conducted as previously reported (1,4,7).

Safety evaluation studies. The solution of **hemoCD-Twins** (14 mM, 200 μL) was injected intraperitoneally to female BALB/cCrSlc mice (9–11 weeks; mean weight: 20 g). After 24 h, mice were anesthetized with isoflurane. Blood samples were collected from the right ventricle and plasma was separated by centrifugation. The measurement of biochemical parameters in plasma samples was performed by Oriental Yeast Industry Co. (Kyoto, Japan). The plasma creatinine levels for mice were assessed using the LabAssay Creatinine (Fujifilm Wako, Japan) according to the manufacturer's instructions. Each organ (brain, heart, lung, spleen, liver, kidney) was collected to confirm no significant damage and no accumulation of **hemoCD-Twins** in the organs at 24 h after the injection. Histopathological analysis for the tissues stained with hematoxylin and eosin (HE) was performed by Sapporo General Pathology Laboratory.

Model of CO and cyanide mixed intoxication in mice. The following animal experiments were carried out under the approval of the Guidelines for Animal Experiments of Doshisha University. The concentration of CO in the atmosphere was adjusted to 5000 ppm, 7500 ppm or 10000 ppm by injecting 20-, 30- or 40-mL pure CO gas (99.999%, Sumitomo Seika Chemicals) to the airtight gas chamber (volume: 4 L) which was filled with air. Female BALB/cCrSlc mice (9–11 weeks; mean weight: 20 g) were exposed to the atmosphere for 5 min, and then removed from the chamber. Mice

were monitored for survival and behavioral recovery under aerobic conditions at room temperature for 40 min. A satisfactory behavioral recovery was considered when lethargic mice returned to the prone position, steady on feet, un-hunched back, and began to walk forward by themselves in the cage (9). For cyanide intoxication, female BALB/cCrSlc mice were orally administered with a NaCN solution (0.1 mg, 0.15 mg, or 0.2 mg NaCN dissolved in 0.1 mL PBS). Animals were observed for survival and behavioral recovery for 45 min after NaCN administration. For CO and cyanide mixed intoxication, female BALB/cCrSlc mice were orally administered with a NaCN solution and placed into the airtight gas chamber. After one minute, pure CO gas (20 mL) was injected into the chamber to reach CO concentration of 5000 ppm. Mice were exposed to the atmosphere for 5 min, and then removed from the chamber. In the CO and HCN mixed intoxication experiments, four out of 70 mice died before taking them out of the chamber containing CO gas and therefore they were not treated with **hemoCD-Twins** or the PBS vehicle control. The surviving animals were immediately given an intraperitoneal injection of **hemoCD-Twins** (200 μL), **hemoCD-P** or **hemoCD-I**. The behavior of mice was monitored under aerobic atmosphere for 24 h.

Models of fire gas poisoning in mice. The following experiments were carried out under the approval of the Guidelines for Animal Experiments of Building Research Institute and Center for Better Living. The instrumental setup (combustion gas toxicity tester N_2, Toyo Seiki Seisaku-sho, Ltd. Japan) was the same as reported previously (12). Male ICR mice (mean weight: 20 g) were placed into spinning wheels in the chamber. Acrylic cloth (mean weight: 17g, 220 mm × 220 mm) was placed into the furnace and heated for 6 min. The temperature at the end of combustion in the furnace was about 260ºC. The combustion gas was first mixed in the pre-mixing chamber and then delivered to the animal exposure chamber. The concentration of the gas components was monitored *in-situ* using Fourier transform infrared (FT-IR) spectroscopy (DX4000, Gasmet, DX4000, Finland) (12). After 7 min, mice were removed from the chamber. Mice that survived this challenge were administered with **hemoCD-Twins** (200 μL) to assess its effect on survival and behavioral recovery rates.

Pharmacokinetic studies in rats. The following experiments were carried out under the approval by the Guidelines for Animal Experiments of Doshisha Women's College of Liberal Arts. Male Wistar rats (300 ± 15 g) were anesthetized with isoflurane. A tube was inserted into the urinary bladder for washing and collecting urine. The solution of **hemoCD-Twins** (14 mM, 2 mL in PBS) was infused into the femoral vein at a rate of 12 mL/h. During and after the infusion, heart rate and blood pressure were continuously monitored using Bio Amps (AD Instruments Ltd). Urine samples were collected at 10 min intervals. At each time point, the bladder was flushed with PBS. The washed PBS and urine were collected from the urinary tract and combined. Samples were analyzed by UV-vis spectroscopy and quantification of oxy-, met-, and CO-complexes were carried out using molar absorption coefficients as previously reported (8). Blood samples were collected from the jugular vein at 0, 10, 20, 30, 40, 50, 60, 75, 90, 120 min time points. Plasma (100 μL) was separated by centrifugation and methanol (50 μL) was added to the plasma to remove serum proteins. After centrifugation, the supernatant (100 μL) was diluted to 1 mL with PBS. CO and **S** were successively added to the solution to transform all the **hemoCD**s to the ferrous-CO complex. Then the solutions were analyzed by size exclusion chromatography (NGC Chromatography System, Bio-Rad Laboratories Inc, attached with an ENrich SEC650 10×300 column, flow rate: 0.5 mL/min with 0.05 M phosphate buffer at pH 7.0 and 4°C. Elution was monitored at 422 nm). Noncompartmental analysis of data was performed by Winnonlin professional version 8.3.3. (Pharsight Corporation, Mountain View, CA, USA). The elimination rate constant, k_{el}, was determined by a linear regression of at least four or five data points from the terminal portion (50 or 60-120 min) of the plasma concentration-time plots. The area under the curve (AUC) was calculated using the linear trapezoidal rule up to the last measured plasma concentration, $C_{p(last)}$, and extrapolated to infinity by addition of the collection term, $C_{p(last)}/k_{el}$. The half-life, $t_{1/2}$, was determined by dividing ln2 by k_{el}. The total plasma clearance, CL_{tot}, was determined by dividing the dose by the AUC. Then, renal clearance (CL_r) was calculated by CL_{tot} multiplying cumulative amount ratio excreted into urine of **hemoCD-Twins** in 2 h**.** Values are expressed as mean±S.D.

Model of non-lethal CO intoxication in rats. The following animal experiments were carried out under the approval by the Institutional Review Board of Tokai University. The experimental setup was the same as previously described (10). Briefly, Sprague-Dawley rats (300 ± 15 g) were anesthetized with 3% sevoflurane and, a tracheal tube was inserted for mechanical ventilation (Rodent Ventilator, Ugo Basile; ventilation rate: 55 per minute). A cannula was inserted into the carotid artery for collecting blood samples. An indwelling catheter was placed in the tail vein and saline was administered at 3 mL/h. Body temperature was maintained at 36.5 ± 0.5°C with a water blanket (MEDI-Therm II, Gaymer Industries Inc.). Rats were then anesthetized with an intraperitoneal injection of pentobarbital and sevoflurane anesthesia was terminated. The mixed CO gas at 2000 ppm in air (flow: 1 L/min, TOMOE SHOKAI Co., LTD) was inhaled for 15 min via the ventilator. After CO exposure, rats were returned to air ventilation and **hemoCD-Twins** (2 mL), **hemoCD-P**, **hemoCD-I**, or PBS were infused from the tail vein at a rate of 15 mL/h. Blood samples were collected at 0, 15, 25, 35, and 45 min and immediately analyzed using a blood gas analyzer, ABL825 (Radiometer Co. Ltd.), which measures CO-Hb% based on a 128 wavelengths spectrometer with a measuring range from 478 to 672 nm. To monitor heart rate and mean blood pressure, one carotid artery was cannulated to the anesthetized rat. The heart rate and blood pressure were monitored using Bio Amps (AD Instruments Ltd).

Blood cyanide and lactate measurements (mice/rats). Blood cyanide concentrations were quantified by a microdiffusion method (11). Samples (mixture of whole blood and water, 1:1, *v*/*v)* were placed into plastic test tubes. In the tubes, micro tubes containing 0.1 mol/L NaOH were placed at the bottom. Then 10% H_3PO_4 solution was added to the sample solution. The tubes were immediately sealed to prevent outer diffusion of HCN. The tubes were shaken for 2 h at room temperature. During the shaking, cyanide ion diffused as HCN from the sample solution and trapped by the NaOH solution in the micro tubes. The cyanide concentration in the NaOH solution was quantified by a spectrophotometric assay following the König reaction using a pyridine-pyrazolone reagent. Calibration curves were prepared using KCN dissolved in 0.1 M NaOH. Significant variability of cyanide concentration in blood was found among individual rats

5 min after oral administration. Therefore, statistical significance was evaluated by comparing the decrease of cyanide in blood from 5 to 45 min time points in the same group. Blood lactate levels were measured by the Lactate Pro2 kit (LT-1730, Arkray, Japan).

Statistical analysis.

Statistical analyses were performed using GraphPad Prism, Version 8.0 (GraphPad Software). All data represent the means ± standard error from at least three different experiments and were analyzed by Student's t-test. Survival curves were analyzed using Kaplan-Meier curves and log-rank test. Differences with *P* values of less than 0.05 were considered significant.

References for Materials and Methods

1) K. Watanabe, H. Kitagishi, K. Kano, Supramolecular ferric porphyrins as cyanide receptors in aqueous solution. *ACS Med. Chem. Lett.* **2**, 943–947 (2011).

2) K. Kano, H. Kitagishi, M. Kodera, S. Hirota, Dioxygen binding to a simple myoglobin model in aqueous solution. *Angew. Chem. Int. Ed.* **44**, 435–438 (2005).

3) K. Kano, H. Kitagishi, S. Tamura, A. Yamada, Anion binding to a ferric porphyrin complexed with per-*O*-methylated β-cyclodextrin in aqueous solution. *J. Am. Chem. Soc.* **126**, 15202–15210 (2004).

4) K. Kano, S. Chimoto, M. Tamaki, Y. Itoh, H. Kitagishi, Supramolecular dioxygen receptors composed of an anionic water-soluble porphinatoiron(**II**) and cyclodextrin dimers. *Dalton Trans.* **41**, 453–461 (2012).

5) K. Kano, H. Kitagishi, M. Kodera, S. Hirota, Dioxygen binding to a simple myoglobin model in aqueous solution. *Angew. Chem. Int. Ed.* **44**, 435–438 (2005).

6) K. Kano, H. Kitagishi, T. Mabuchi, M. Kodera, S. Hirota, A myoglobin functional model composed of a ferrous porphyrin and a cyclodextrin dimer with an imidazole linker. *Chem. Asian J.* **1**, 358–366 (2006).

7) H. Kitagishi, S. Minegishi, A. Yumura, S. Negi, S. Taketani, Y. Amagase, Y. Mizukawa, T. Urushidani, Y. Sugiura, K. Kano, Feedback response to selective depletion of endogenous carbon monoxide in the blood. *J. Am. Chem. Soc.* **138**, 5417–5425 (2016).

8) H. Kitagishi, S. Negi, A. Kiriyama, A. Honbo, Y. Sugiura, A. T. Kawaguchi, K. Kano, A diatomic molecule receptor that removes CO in a living organism. *Angew. Chem. Int. Ed.* **49**, 1312–1315 (2010).

9) D. L. Crankshaw, D. J. W. Goon, J. E. Briggs, D. DeLong, M. Kuskowski, S. E. Patterson, H. T. Nagasawa, A novel paradigm for assessing efficacies of potential antidotes against neurotoxins in mice. *Toxicol. Lett.* **175**, 111–117 (2007).

10) Q. Mao, A. T. Kawaguchi, S. Mizobata, R. Motterlin, R. Foresti, H. Kitagishi, Sensitive quantification of carbon monoxide in vivo reveals a protective role of circulating hemoglobin in CO intoxication. *Commun. Biol*. **4**, 425 (2021).

11) Y. Okada, H. Miyaguchi, Development of a handy microdiffusion device using two plastic test tubes for accurately quantifying cyanide in blood. *Forensic Toxicol.* **38**, 542–546 (2020).

12) X. Zhao, H. Yoshioka, T. Noguchi, S. Fujimoto, Y. Tanaike, T. Hayakawa, Y. Hase, T. Naruse, Fundamental study of gas toxicity with respect to fire stages. *Fire Sci. Tech.* **36**, 11–24 (2017).

Table S1. O_2 and CO binding parameters for ferrous iron(II) **hemoCD-P**, **hemoCD-I**, and heme proteins.[a]

	k_{on}^{O2} ($M^{-1}s^{-1}$)	k_{off}^{O2} (s^{-1})	K^{O2} (M^{-1})	k_{on}^{CO} ($M^{-1}s^{-1}$)	k_{off}^{CO} (s^{-1})	K^{CO} (M^{-1})	K^{CO}/K^{O2} (= M)
**hemoCD-P**[1–3]	4.7×10^7	800	5.9×10^4	1.3×10^7	2.5×10^{-4}	5.2×10^{10}	8.9×10^5
**hemoCD-I**[3,4]	–	–	3.5×10^5	–	–	4.9×10^8	1.4×10^3
Hb-R (α/β)[5]	(3.6–7.6) $\times 10^7$	16–32	(2.3–2.5) $\times 10^6$	(6.0–7.4) $\times 10^6$	0.007–0.012	(5–10) $\times 10^8$	200–450
Hb-T (α/β)[5]	(5.6–6.7) $\times 10^6$	430–670	(1.0–1.3) $\times 10^4$	(0.7–1.6) $\times 10^5$	0.1	(0.7–1.6) $\times 10^6$	55–160
Mb[6,7]	1.9×10^7	22	8.6×10^5	7.6×10^5	2.2×10^{-2}	3.5×10^7	41
C*c*O[8]	(1.0–6.0) $\times 10^8$	10	(1.0–6.0) $\times 10^7$	(0.7–1.2) $\times 10^5$	2.2×10^{-2}	(3.0–6.0) $\times 10^6$	0.1–0.6
hNgb[8,9]	2.5×10^8	0.8	3.1×10^8	6.5×10^7	1.4×10^{-2}	4.9×10^9	15
$hNgb_{H64Q\text{-}CCC}$[10]	7.2×10^8	18	3.9×10^7	1.6×10^8	4.2×10^{-4}	3.8×10^{11}	9.7×10^3

[a]These parameters were determined in aqueous solutions at ambient temperatures (20-25˚C).

References for Table S1

1) K. Kano, H. Kitagishi, C. Dagallier, M. Kodera, T. Matsuo, T. Hayashi, Y. Hisaeda, S. Hirota, *Inorg. Chem.* **45***,* 4448–4460 (2006).

2) Q. Mao, A. T. Kawaguchi, S. Mizobata, R. Motterlin, R. Foresti, H. Kitagishi, *Commun. Biol.* **4**, 425 (2021).

3) H. Kitagishi, K. Kano, *Chem. Commun*. **57**, 148–173 (2021).

4) K. Kano, H. Kitagishi, T. Mabushi, M. Kodera, S. Hirota, *Chem. Asian J.* **1**, 358–366 (2-006).

5) S. Unzai, R. Eich, N. Shibayama, J. S. Olson, H. Morimoto, *J. Biol. Chem.* **273**, 23150–23159 (1998).

6) B. A. Springer, S. G. Sligar, J. S. Olson, G. N. Phillips, Jr., *Chem. Rev.* **94**, 699–714 (1994).

7) D. A. Moffet, M. A. Case, J. C. House, K . Vogel, R. D. Williams, T. G. Spiro, G. L. McLendon, M. H. Hecht, *J. Am. Chem. Soc*. **123**, 2109–2115 (2001).

8) J. J. Rose, K. A. Bocian, Q. Xu, L. Wang, A. W. DeMartino, X. Chen, C. G. Corey, D. A. Guimarães, I. Azarov, X. N. Huang, Q. Tong, L. Guo, M. Nouraie, C. F. McTiernan, C. P. O'Donnell, J. Tejero, S. Shiva, M. Gladwin. *J. Biol. Chem.* **295**, 6357–6371 (2020).

9) S. Dewilde, L. Kiger, T. Burmester, T. Hankeln, V. Baudin-Creuza, T. Aerts, M.C. Marden, R. Caubergs, L. Moens, *J. Biol. Chem*. **276**, 38949–38955 (2001).

10) I. Azarov, L. Wang, J. J. Rose, Q. Xu, X. N. Huang, A. Belanger, Y. Wang, L. Guo, C. Liu, K. B. Ucer, C. F. McTiernan, C. P. O'Donnell, S. Shiva, J. Tejero, D. B. Kim-Shapiro, M. T. Gladwin, *Sci. Transl. Med.* **8**, 368ra173 (2016).

Table S2. Cyanide binding parameters for ferric iron(III) **hemoCD-P** and **hemoCD-I**. The binding kinetics of cyanide to heme proteins (met-Hb, C*c*O) and hydroxocobalamin (OHCbl) are shown for comparison.

	k_{on}^{CN} (M^{-1} s^{-1})	k_{off}^{CN} (s^{-1})	K^{CN} (M^{-1})
**hemoCD-P**[1–4]	14.9	7.1×10^{-6}	2.1×10^{6}
**hemoCD-I**[1–4]	193	7.4×10^{-5}	2.6×10^{6}
**hemoCD-I** with serum proteins[1–4]	104	7.8×10^{-5}	1.3×10^{6}
met-Hb[1–4]	320	3.9×10^{-4}	8.1×10^{5}
met-Hb with serum proteins[1–4]	208	6.4×10^{-4}	3.2×10^{5}
OHCbl[1–4]	81.5	1.0×10^{-5}	8.0×10^{6}
OHCbl with serum proteins[1–4]	6.5	5.0×10^{-4}	1.3×10^{4}
C*c*O[5]	5000	5.0×10^{-4}	1.0×10^{7}
C*c*O (oxidized)[6–8]	2.0	2.0×10^{-6}	1.0×10^{6}
C*c*O (reduced)[6–8]	130	6.5×10^{-2}	2.0×10^{3}
C*c*O (partially reduced)[6–8]	2.0×10^{6}	$(1.5–4.7) \times 10^{-2}$	$(0.011–6.7) \times 10^{7}$

References for Table S2

1) These parameters were determined in aqueous solutions at 37°C.
2) K. Watanabe, H. Kitagishi, K. Kano, *ACS Med. Chem. Lett.*, **2**, 943–947 (2011).
3) K. Watanabe, H. Kitagishi, K. Kano, *Angew. Chem. Int. Ed.,* **52**, 6894–6897 (2013).
4) H. Kitagishi, K. Kano, *Chem. Comm.* **57**, 148–173 (2021).
5) C. E. Cooper, G. C. Brown, *J. Bioengerg. BIomembr.* **40**, 533–539 (2008).
6) H. B. Leavsley, L. Li, K. Prabhakaran, J. L. Borowitz, G. E. Isom, *Toxicol. Sci.* **101**, 101–111 (2008).
7) E. Antonini, M. Brunori, C. Greenwood, B. G. Malmstrom, G. Rotilio, *Eur. J. Biochem*. **23**, 396–400 (1971).
8) M. Jones, D. Bickar, M. T. Wilson, M. Brunori, A. Colosimo, and P. Sarti, *Biochem. J.* **220**, 57–66 (1984).

Table S3. Toxicity test for intraperitoneal (i.p.) injection of sodium dithionite (**S**) to mice.[a]

Dose of sodium dithionite (**S**) (mg/kg)	*n*	Alive	Dead
250	3	3	0
500	3	3	0
750	4	4	0
1000	4	0	4
1500	4	0	4

[a]Solutions of sodium dithionite in PBS (0.2 mL) were injected at increasing doses in mice (body weights = 20 ± 2 g).

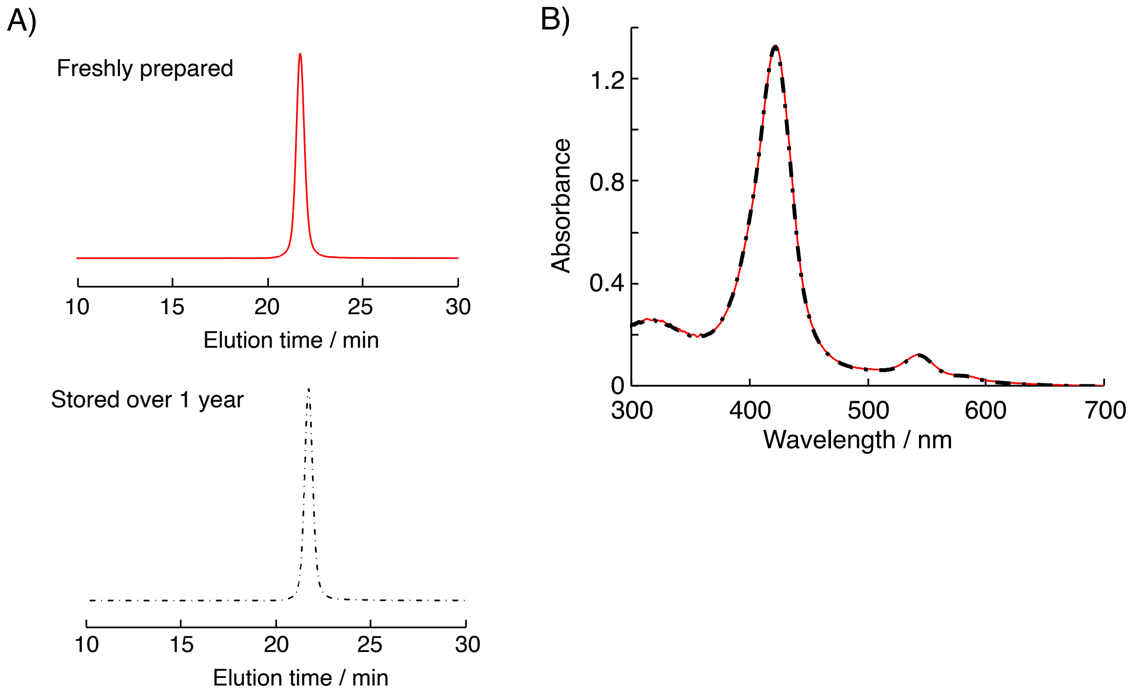

Fig. S1. Size exclusion chromatography (SEC) and UV-vis analyses to assess the chemical stability of **hemoCD-Twins**. (A) SEC traces of a solution of **hemoCD-Twins** in the ferric iron(III) state immediately after preparation (top) and from a solution stored over one year at room temperature without light shielding (bottom). The elution time was monitored at 280 nm. (B) Overlaid UV-vis spectra of the diluted solutions of **hemoCD-Twins**. The stock solution (**F** = 3.5 mM, **P** = **I** = 4 mM in PBS) stored at room temperature over a year was reduced by adding **S** (5 mg/mL) followed by dilution from 5 μL to 3 mL in air-saturated PBS (black dotted line). The spectrum obtained from a freshly prepared stock solution (red line) is shown for comparison. The identical traces in A and B indicate the high chemical stability of **hemoCD-Twins** in the ferric iron(III) state.

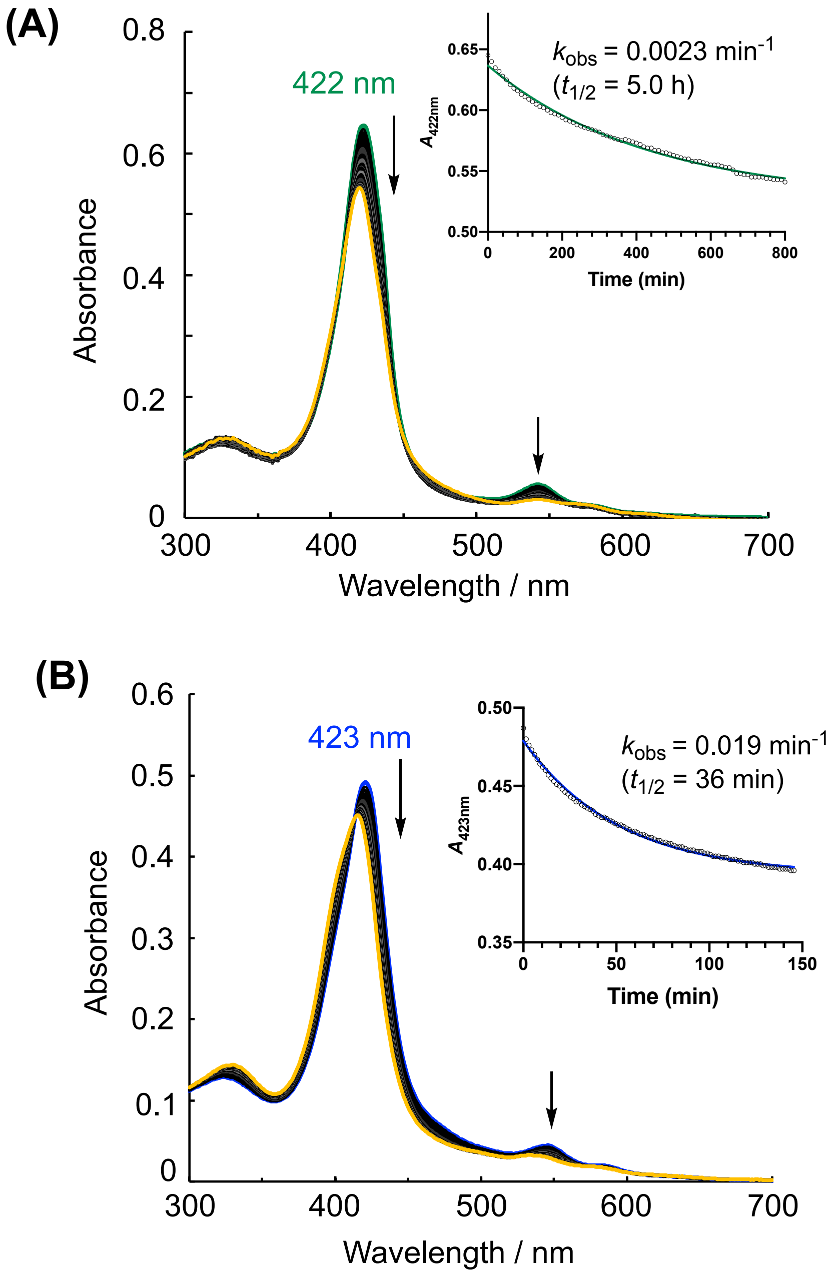

Fig. S2. Autoxidation reaction of the O_2-adducts of **hemoCD-P** (**A**) and **hemoCD-I** (**B**) dissolved in phosphate buffered saline (PBS). The spectral measurements were performed at 37°C. Insets show the time course of the absorbances. The solid lines are the best fit to the experimental data using single exponential kinetic model to give the first-order rate constants (k_{obs}), which are in good agreement with those observed in the mixed system **hemoCD-Twins** shown in Fig. 1A in the main text.

(A)

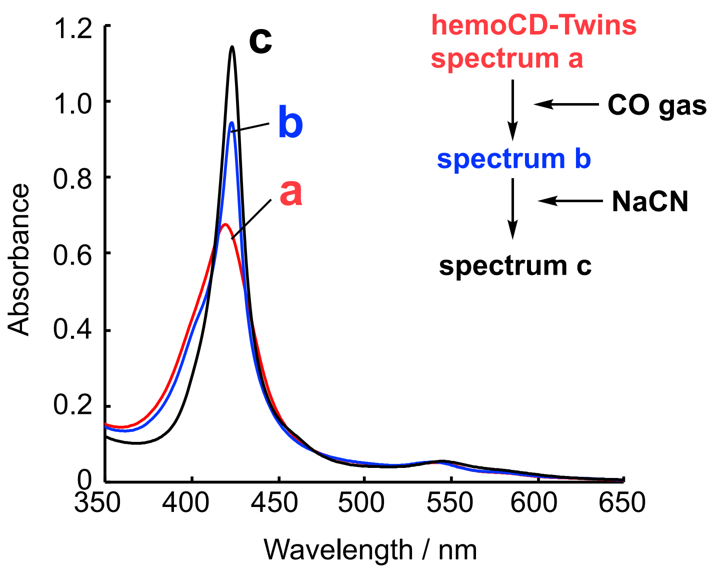

(B)

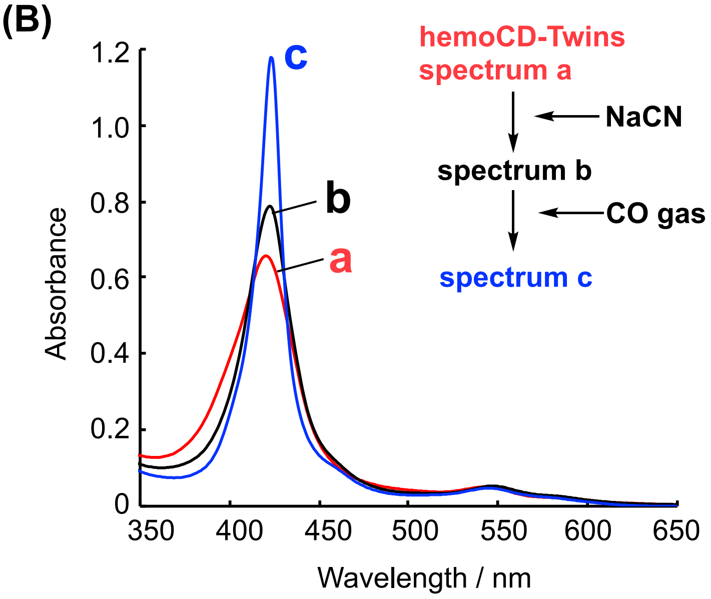

Fig. S3. UV-vis spectral changes of **hemoCD-Twins** (3.5 mM, 4 μL) diluted in air-saturated PBS (3 mL) before and after the addition of excess CO and NaCN. The spectra were changed upon addition of NaCN and CO regardless of whether CO gas (**A**) or NaCN (**B**) were added first, and the final spectra c were the same. This experiment demonstrates that **hemoCD-Twins** is reactive to both CO and cyanide regardless of the order of the addition.

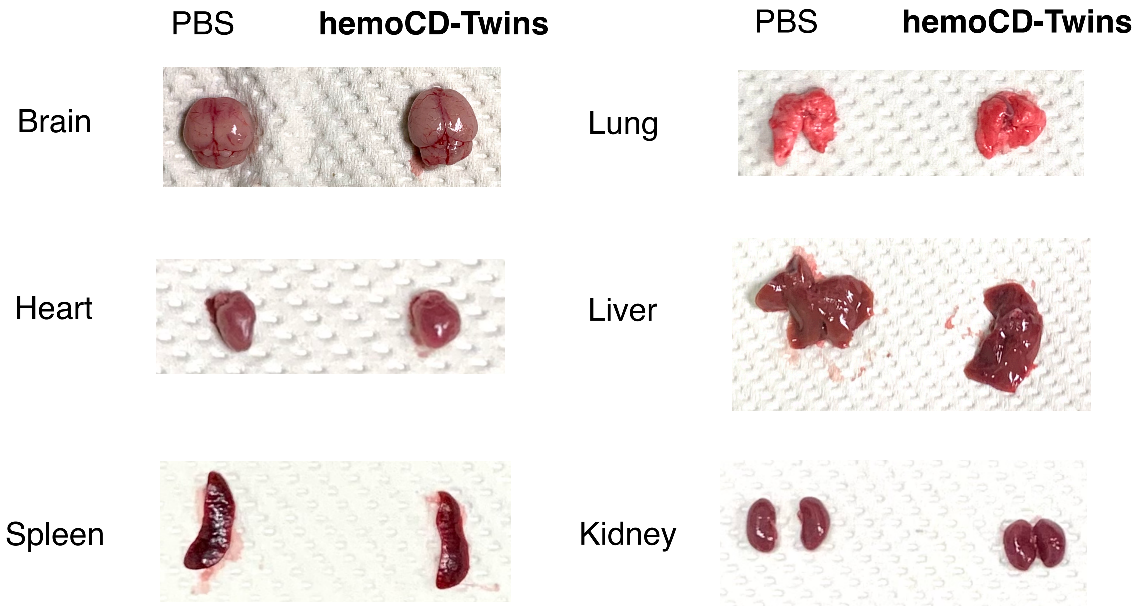

Fig. S4. Appearance of brain, lung, heart, liver, spleen, and kidney collected from mice 24 h after intraperitoneal administration of PBS (left) or **hemoCD-Twins** (right, 14 mM in PBS, 0.2 mL). Little accumulation of **hemoCD-Twins** and no visible damage in these organs were detected after administration of the compound.

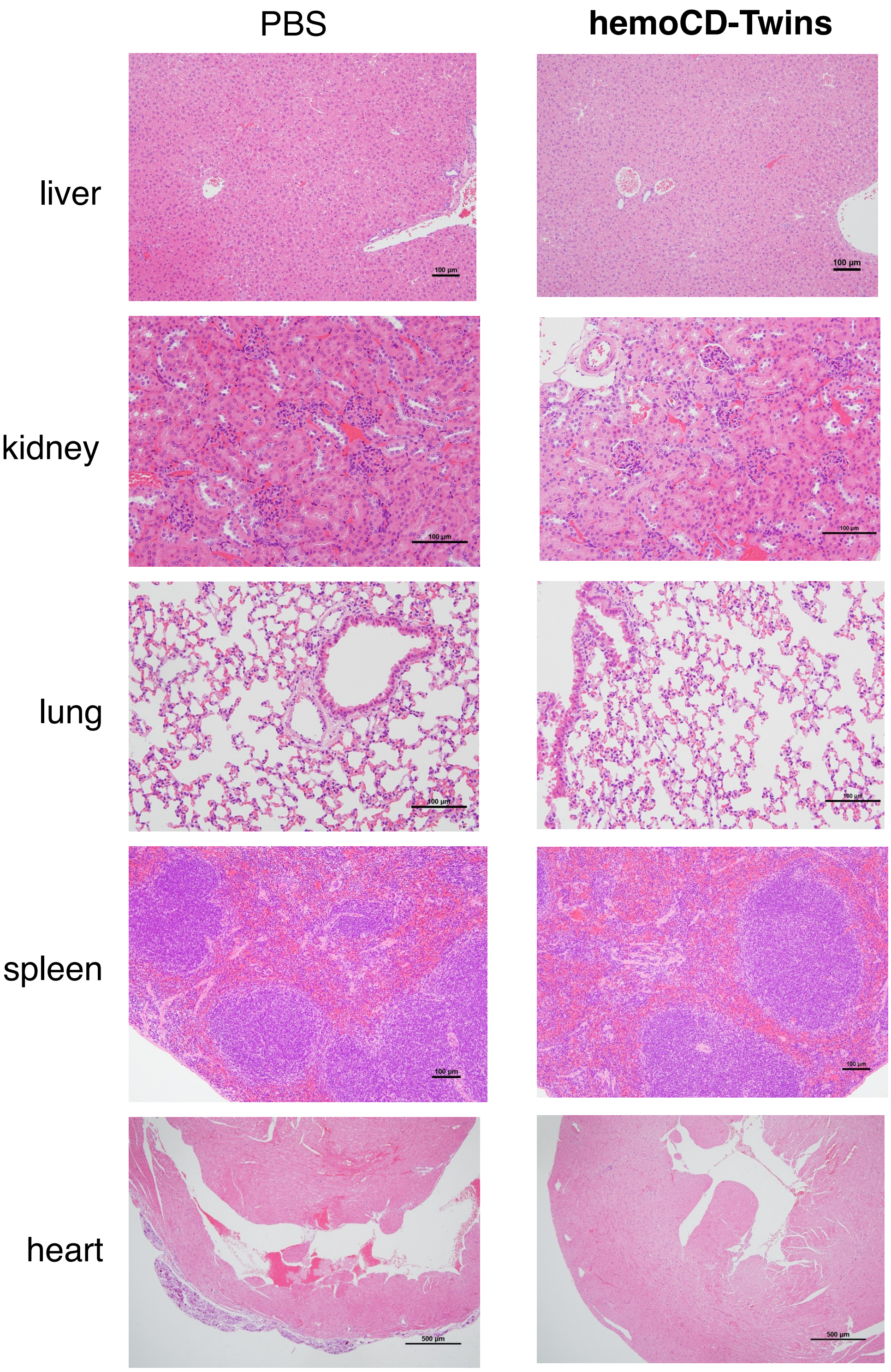

Fig. S5. Histological analysis of liver, kidney, lung, spleen and heart tissues collected from mice at 24 h after i.p. administration of PBS (left, 0.2 mL) or **hemoCD-Twins** (right, 14 mM in PBS, 0.2 mL). The tissues stained with hematoxylin and eosin (HE) revealed no abnormalities after **hemoCD-Twins** administration.

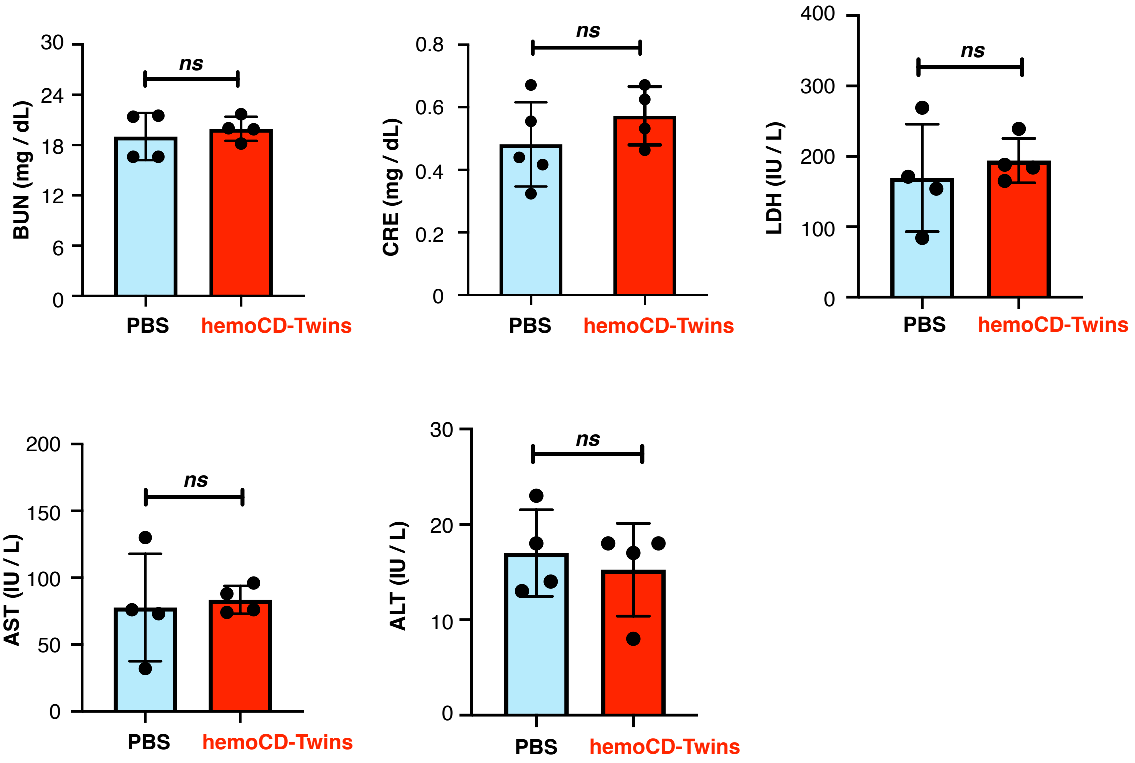

Fig. S6. Effect of **hemoCD-Twins** on biochemical markers indicative of kidney and liver function in mice. Plasma samples were collected from mice 24 h after an i.p. injection of **hemoCD-Twins** (14 mM, 0.2 mL) or PBS (0.2 mL). Blood urea nitrogen (BUN), creatinine (CRE), aspartate aminotransferase (AST), alanine aminotransferase (ALT), and lactate dehydrogenase (LDH) were then measured as reported in Materials and Method. Statistical significance: *ns*, not significant.

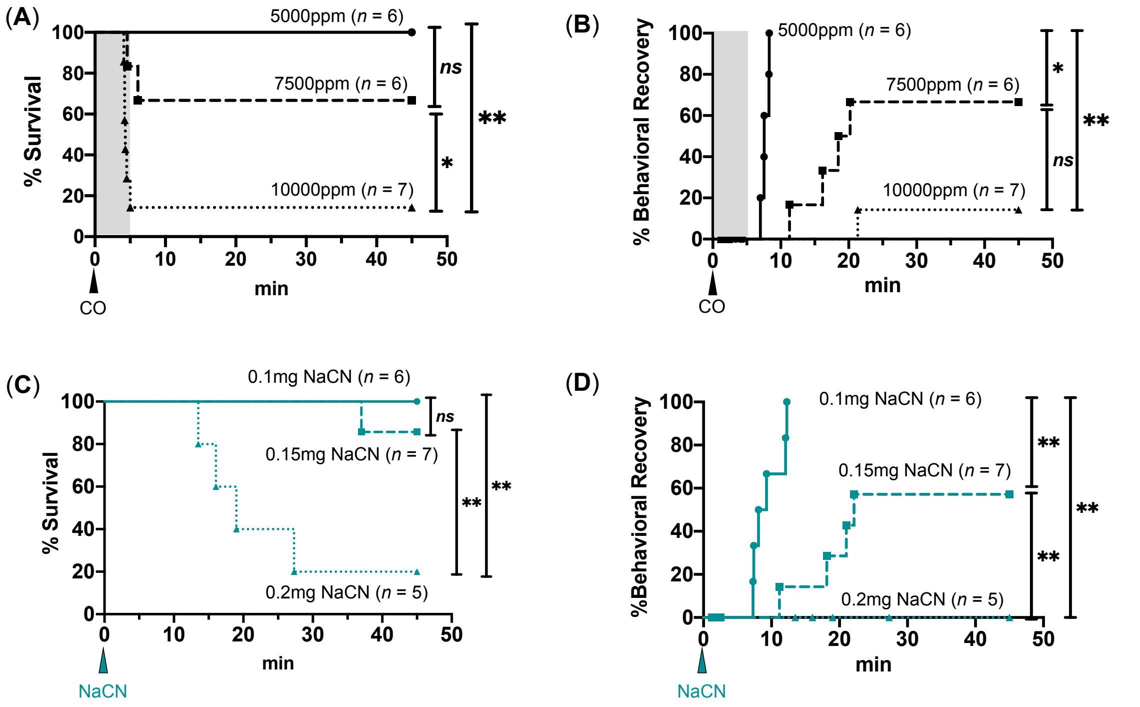

Fig. S7. Dose dependent effects of CO and NaCN in mice. Toxic effects of CO gas (**A**,**B**) and NaCN (**C**,**D**) were evaluated by monitoring survival rates (**A**,**C**) and behavioral recovery (**B**,**D**). Statistical significance: *$p < 0.05$, **$p < 0.01$; *ns*, not significant.

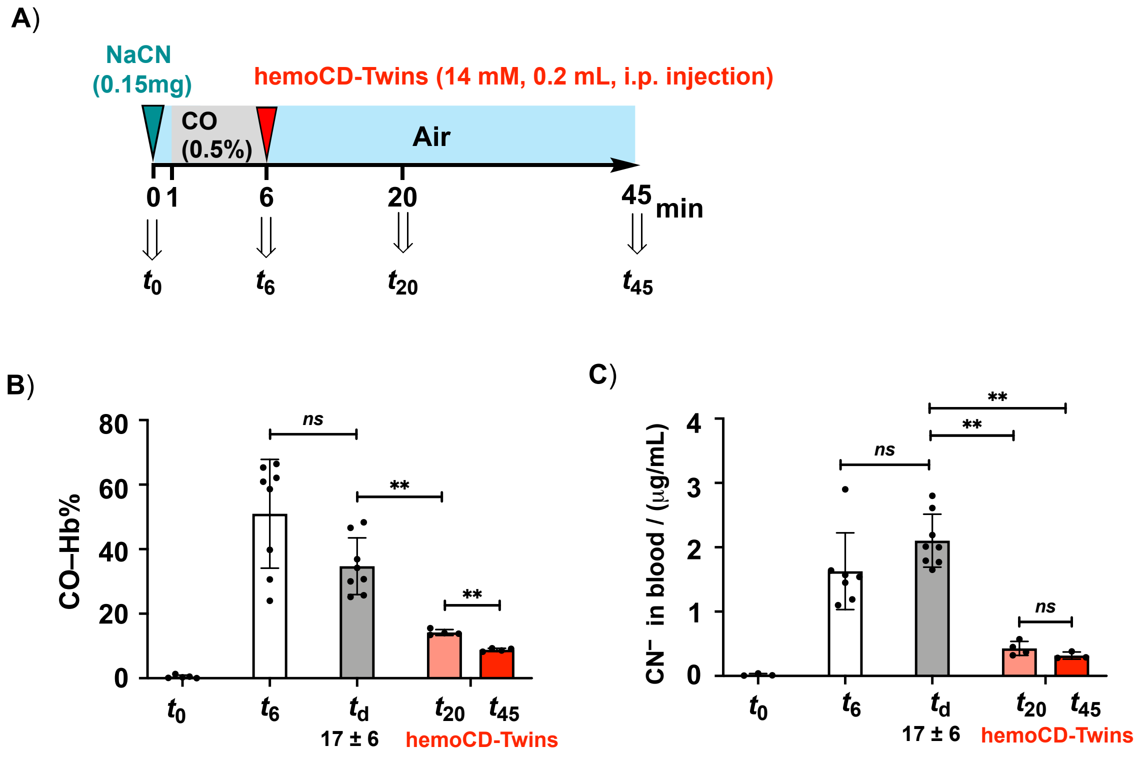

Fig. S8. Changes in CO-Hb levels (%) and cyanide concentrations (μg/mL) in blood of mice exposed to CO and cyanide in the presence or absence of **hemoCD-Twins**. Mice were first orally administered with NaCN in PBS (0.15 mg) and then exposed to 5000 ppm CO atmosphere for 5 min. At 6 min after exposure (t_6), **hemoCD-Twins** was administered and blood samples were collected at different time points as indicated in (**A**). t_d (17 ± 6) corresponds to the time when mice were dead. Statistical significance: *p < 0.05, **p < 0.01; *ns*, not significant.

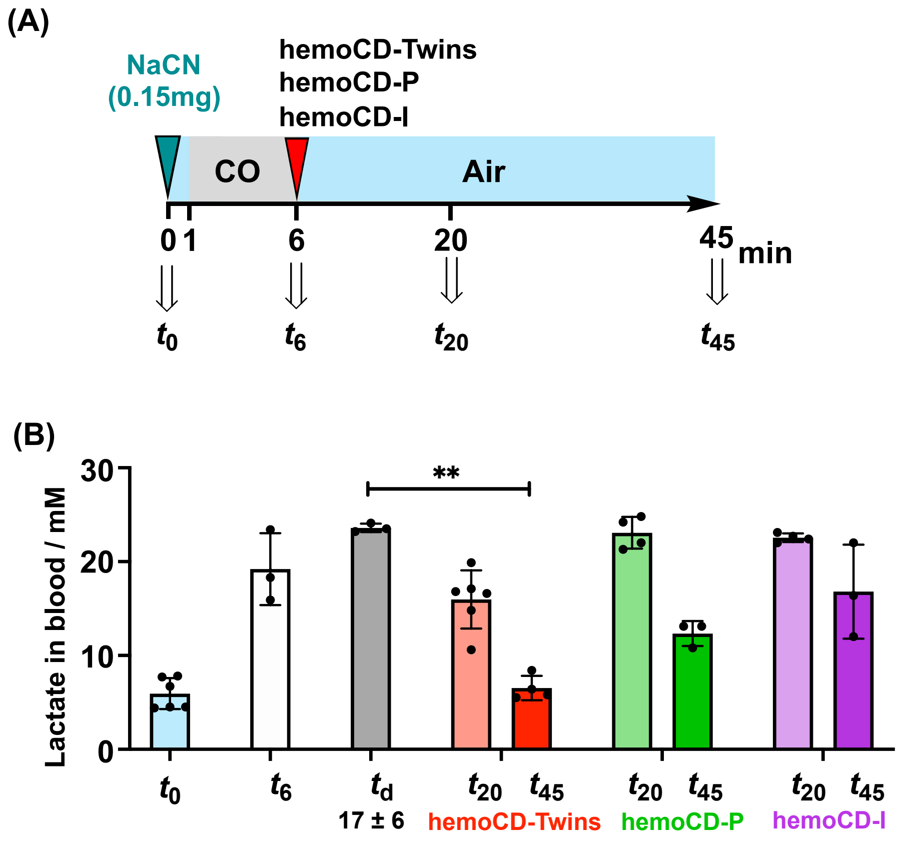

Fig. S9. Lactate concentrations in blood of mice exposed to CO and cyanide in the presence or absence of **hemoCD**s. Mice were first orally administered with NaCN in PBS (0.15 mg) and then exposed to 5000 ppm CO atmosphere for 5 min. At 6 min after exposure (t_6), **hemoCD-Twins** (14 mM), **hemoCD-P** (7 mM) or **hemoCD-I** (7 mM) were injected i.p., and blood samples were collected at different time points as indicated in (**A**). t_d (17 ± 6) corresponds to the time when mice were dead. The data show significant blood acidification upon CO and cyanide mixed poisonings and effective recovery of basal lactate levels by **hemoCD-Twins** treatment. Statistical significance: *p < 0.05, **p < 0.01; *ns*, not significant.

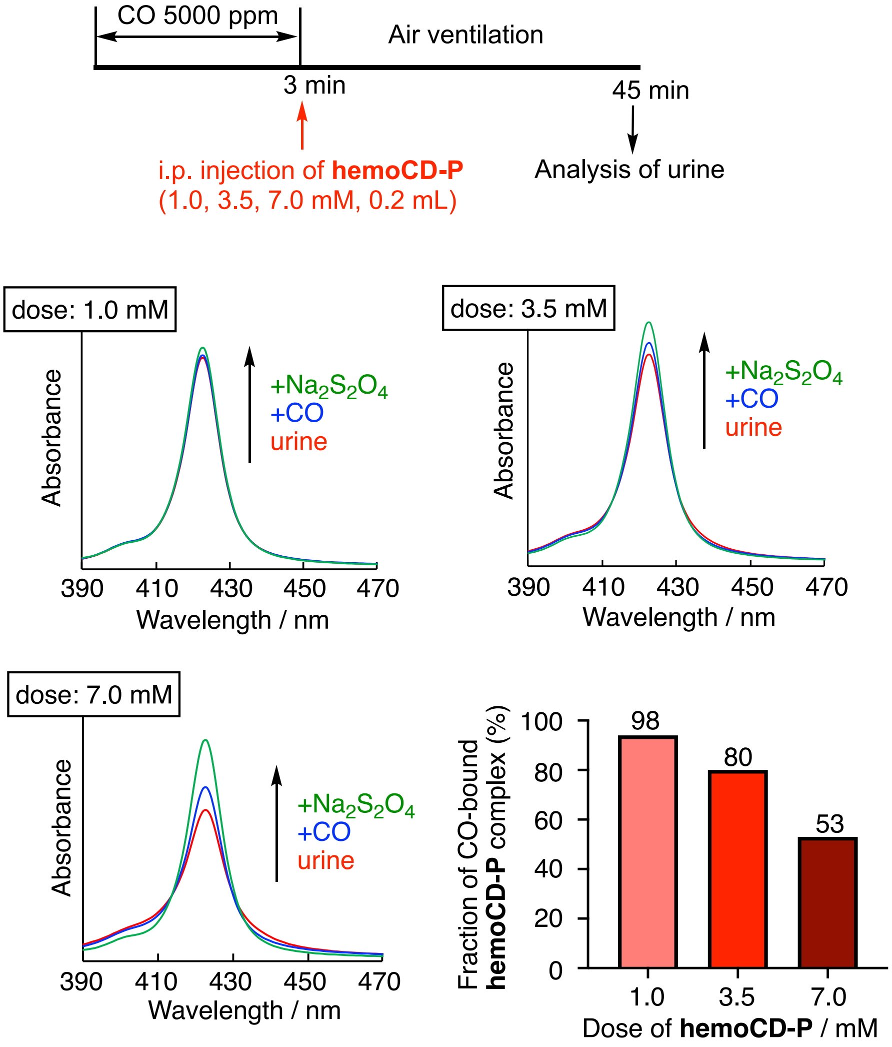

Fig. S10. Dose dependent effect of **hemoCD-P** on its capacity to bind CO in mice. Mice exposed to CO gas (5000 ppm) for 3 min were administered with **hemoCD-P** at different concentrations. Urine samples were collected over 45 min, then analyzed by UV-vis spectroscopy, resulting in 40–60% of the injected **hemoCD-P** recovered in urine. The fraction (%) of the CO-complex of **hemoCD-P** contained in urine were quantified using a molar absorption coefficient reported elsewhere (*J. Am. Chem. Soc.* **138**, 5417–5425, 2016). The results demonstrate that 7.0 mM **hemoCD-P** is sufficient to neutralize CO in mice exposed to CO gas because there is still enough compound in the circulation with the capacity to bind additional CO.

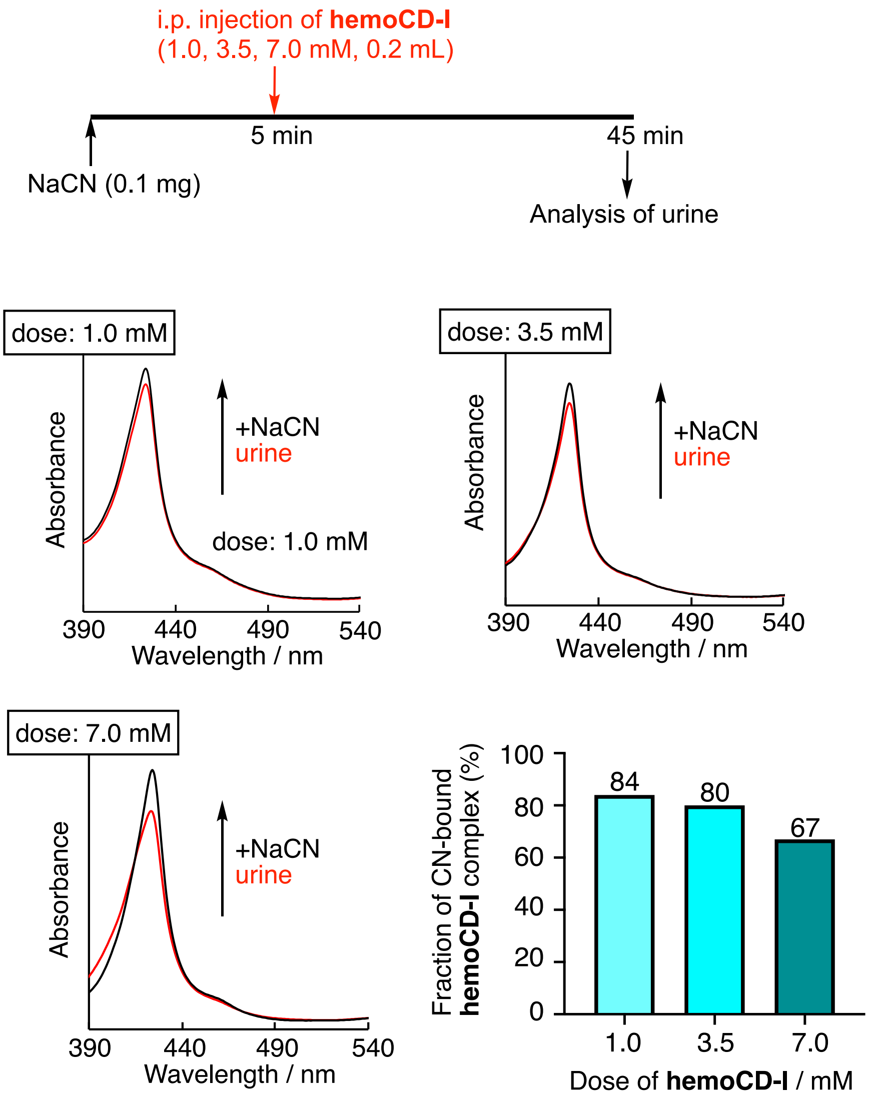

Fig. S11. Dose dependent effect of **hemoCD-I** on its capacity to bind cyanide in mice. Mice orally treated with NaCN (0.1 mg) were administered with **hemoCD-I** at different concentrations. Urine samples were collected over 45 min, then analyzed by UV-vis spectroscopy, resulting in 40–60% of the injected **hemoCD-I** recovered in urine. The fraction (%) of cyanide-complex of **hemoCD-I** contained in urine were quantified using a molar absorption coefficient reported elsewhere (*ACS Med. Chem. Lett.* **2**, 943–947, 2011). The results demonstrate that 7.0 mM **hemoCD-I** is sufficient to neutralize the cyanide in mice exposed to NaCN because there is still enough compound in the circulation with the capacity to bind additional cyanide.

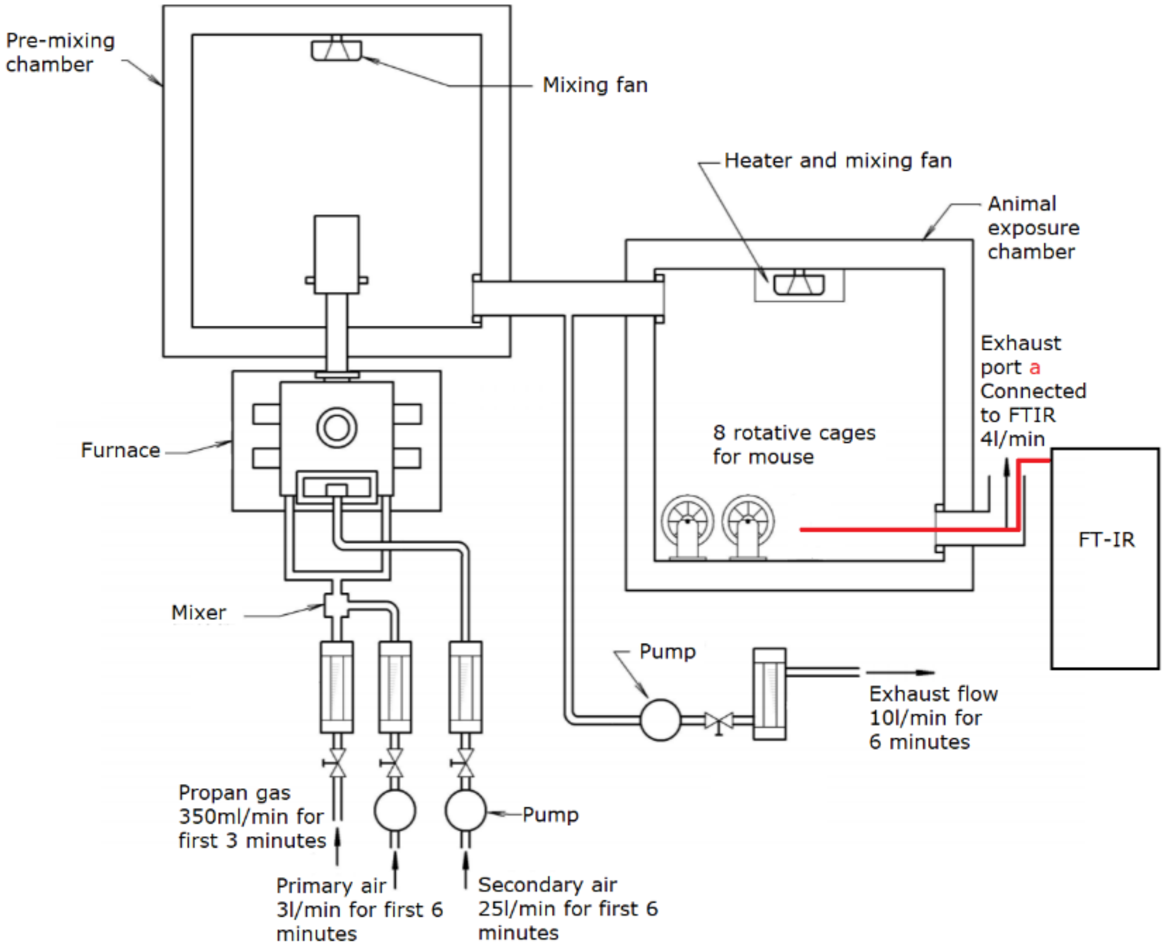

Fig. S12. Schematic configuration of the combustion gas toxicity tester set at Tsukuba Building Research and Testing Laboratory. A picture of this apparatus is shown in Fig. 4A in the main text.

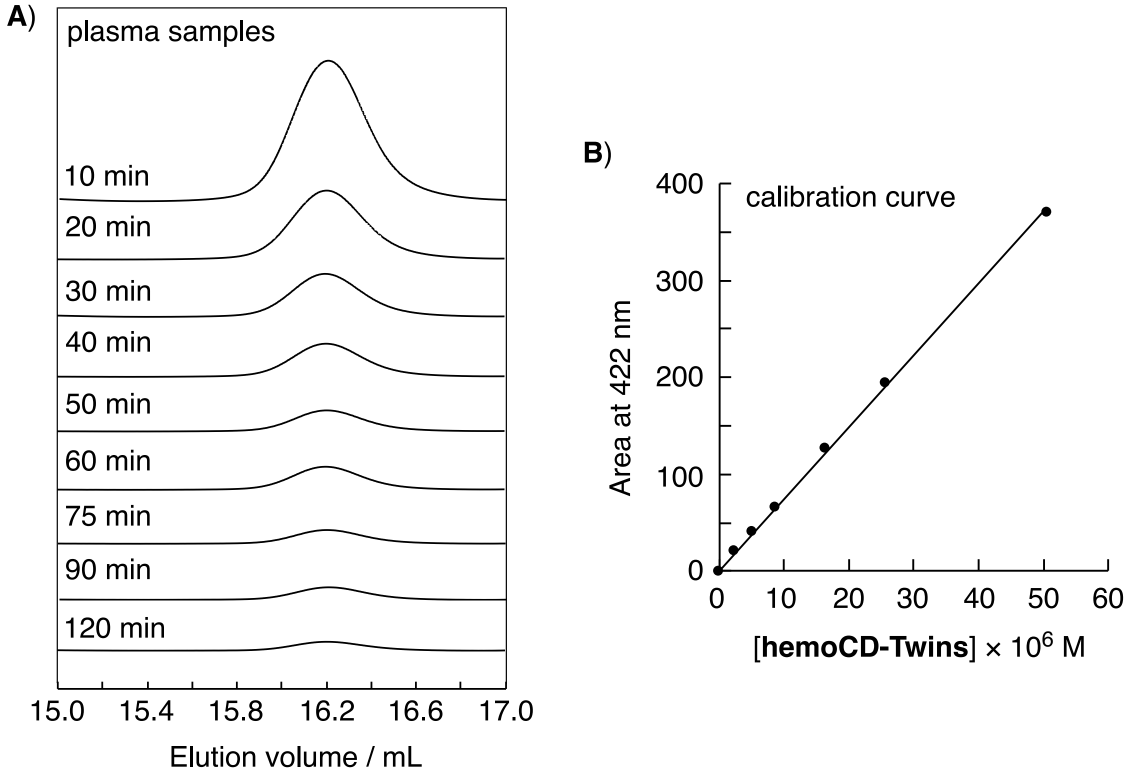

Fig. S13. Quantification of **hemoCD-Twins** in rat plasma. (A) Size exclusion chromatograms of the plasma samples obtained during and after an i.v. infusion of **hemoCD-Twins** (14 mM in PBS, 2 mL, infused at the rate of 12 mL/h for 10 min). A ENrich 650 10×300 column was used and elution was performed at a flow rate of 0.5 mL/min with 0.05M phosphate buffer at pH 7.0 and 4 ºC. Elution was monitored at 422 nm. (B) A calibration curve (peak area vs **hemoCD-Twins** at various concentrations) for the size exclusion chromatograms.

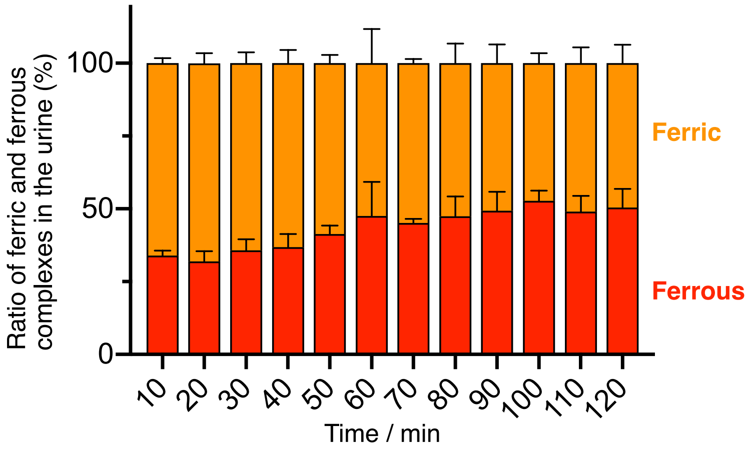

Fig. S14. Ferric/ferrous ratio of **hemoCD-Twins** excreted in rat urines during and after an i.v. infusion of **hemoCD-Twins** (14 mM in PBS, 2 mL, infused at the rate of 12 mL/h for 10 min). Absorption spectra of the samples were measured after appropriate dilution with PBS. The absorbances at 422 nm were recorded before and after successive additions of CO and excess $Na_2S_2O_4$. Based on the absorbance values, the ratio of the ferric and ferrous complexes as well as the total amount of **hemoCD-Twins** (ferric plus ferrous) were quantified with the same method for mouse (Fig. 2 in the main text).

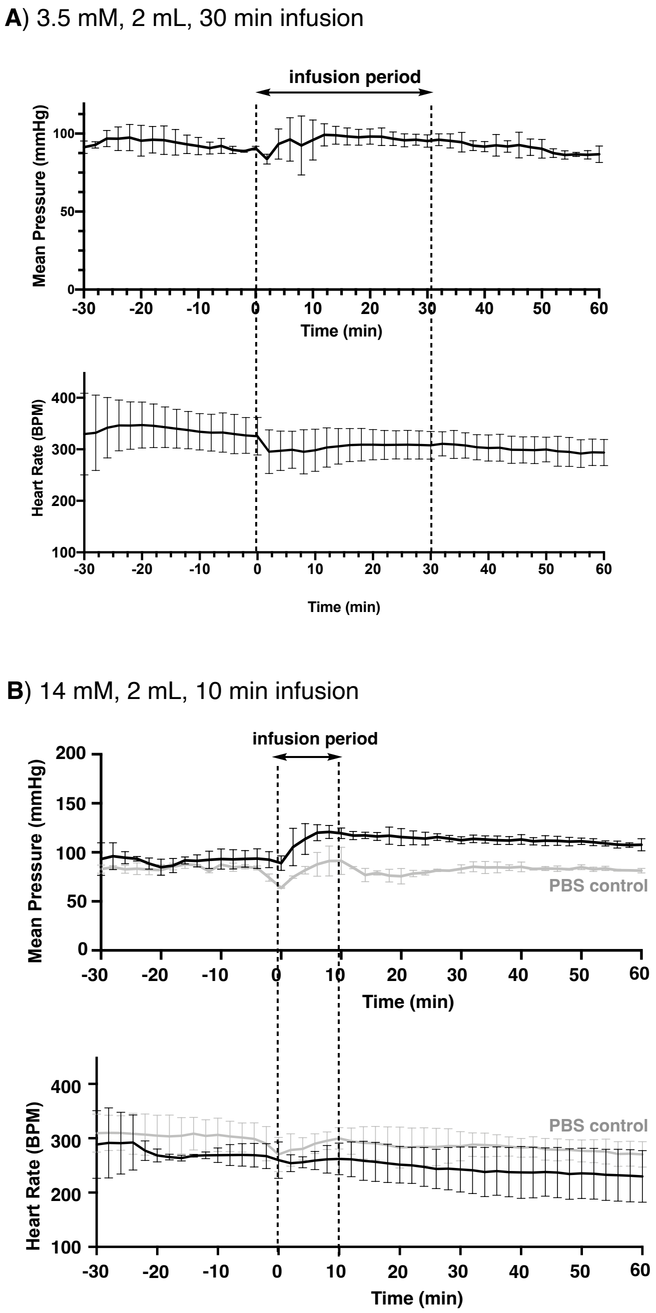

Fig. S15. Mean arterial blood pressure and heart rate in anesthetized rats following intravenous infusions of **hemoCD-Twins** in PBS at 3.5 mM (**A**) or 14 mM (**B**) (2 mL infused at the rate of 4 or 12 mL/h). The temporary increase in blood pressure observed in (**B**) is ascribed to the rapid intravenous infusion, as similarly observed in the PBS control (2 mL, 10 min infusion). Each plot represents the mean±SD (*n* = 3).

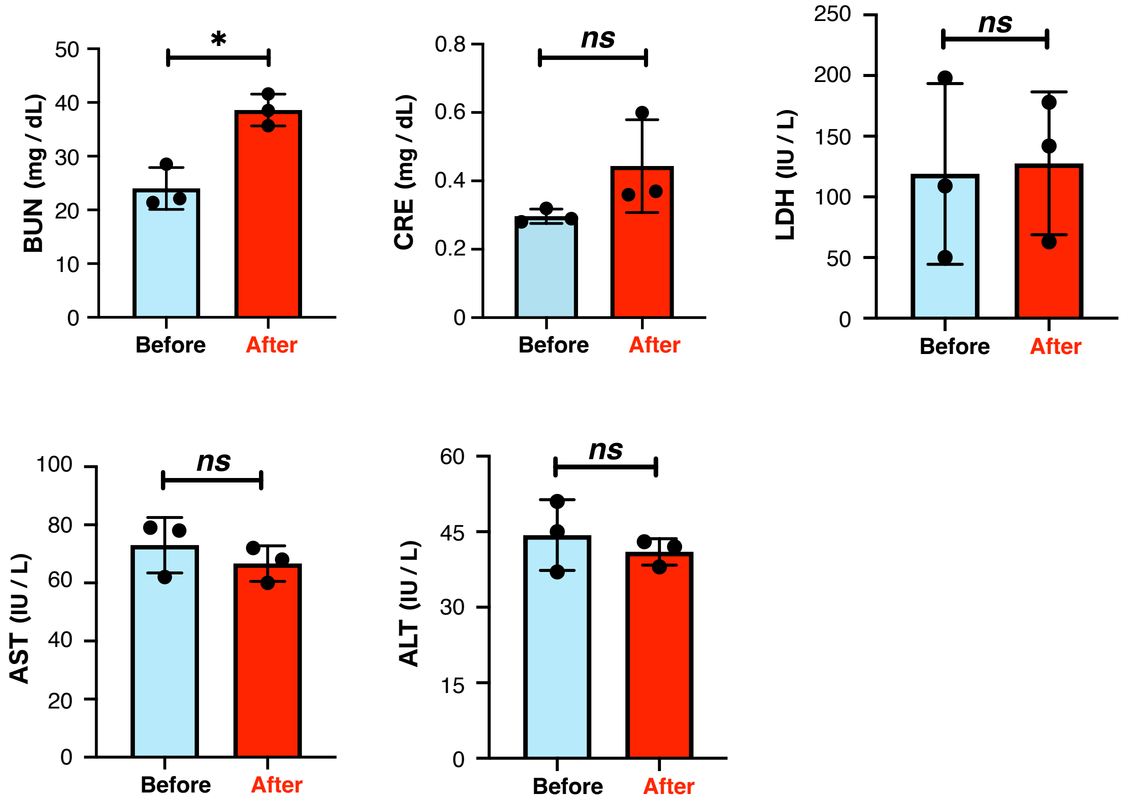

Fig. S16. Effect of **hemoCD-Twins** on biochemical markers indicative of kidney and liver function in rats before and 3 h after the infusion of **hemoCD-Twins** (14 mM in PBS, infused for 10 min at the rate of 12 mL/h). Blood urea nitrogen (BUN), creatinine (CRE), lactate dehydrogenase (LDH), alanine aspartate aminotransferase (AST), aminotransferase (ALT) were then measured as reported in Materials and Methods. Statistical significance: *$p < 0.05$, **$p < 0.01$; *ns*, not significant.

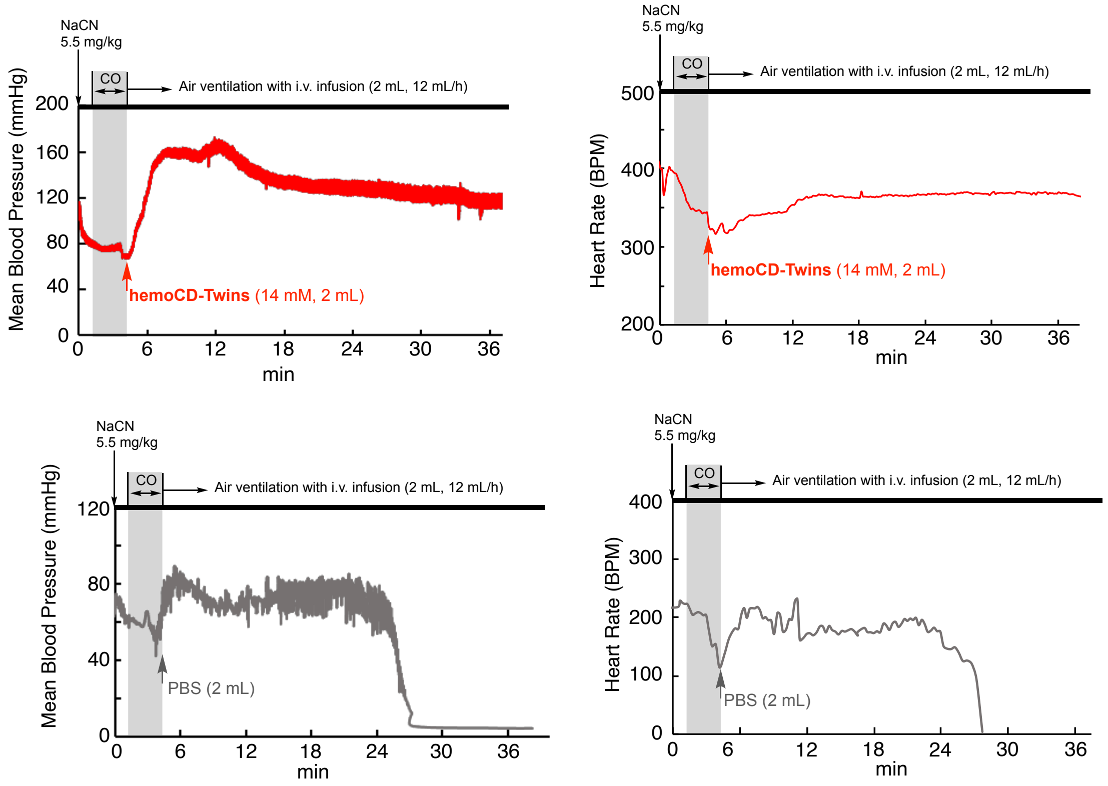

Fig. S17. Changes in hemodynamic parameters in rats after infusion of **hemoCD-Twins**. Time course of mean blood pressure and heart rate were monitored in rats after successive challenge with NaCN (5.5 mg/kg, oral admin), CO gas (5000 ppm inhalation for 3 min) followed by i.v. infusion of **hemoCD-Twins** (14 mM, 2 mL in PBS, infused at the rate of 12 mL/h) or PBS (control vehicle) supported by air ventilation.
